# Supplementary material for: A Systematic Review and Meta-Analysis of MicroRNA as Predictive Biomarkers of Acute Kidney Injury
Source: Biomedicines. 2024 Jul 30;12(8):1695. doi: 10.3390/biomedicines12081695 (PMC11351452; doi:10.3390/biomedicines12081695)
Supplement: Supplementary file 1 [file biomedicines-12-01695-s001.zip › File S1.pdf]

File S1: NEWCASTLE - OTTAWA QUALITY ASSESSMENT SCALE

CASE CONTROL STUDIES

Note: A study can be awarded a maximum of one star for each numbered item within the Selection and Exposure categories. A maximum of two stars can be given for Comparability. Overall maximum points is 9 for any individual study.

Selection: (Maximum 4 stars)

1) Is the case definition adequate?
   a) uses validated measurement tool for diagnosing AKI (i.e. KDIGO, AKIN or RIFLE criteria). *
   b) does not use independently validated criteria.
   c) no description

2) Representativeness of the cases
   a) consecutive or obviously representative series of cases *
   b) potential for selection biases or not stated

3) Selection of Controls
   a) community controls or same community as the AKI cohort *
   b) hospital controls
   c) no description

4) Definition of Controls
   a) no history of AKI *
   b) no description of source

Comparability: (Maximum 2 stars)

1) Comparability of cases and controls on the basis of the design or analysis
   a) study controls for baseline demographics (age/sex). *
   b) study controls for comorbidities (i.e. DM, HTN, CKD). *
   c) study not controlled for relevant confounders or information not provided.

Exposure: (Maximum 3 stars)

1) Ascertainment of exposure
   a) Objective validated laboratory method (i.e. qRT-PCR/microarray/sequencing). *
   b) Uses non-standard or non-validated laboratory methods.
   c) No description/non-standard laboratory methods used.

2) Same method of ascertainment for cases and controls
   a) yes *
   b) no

3) Non-Response rate
   a) same rate for both groups *
   b) non respondents described
   c) rate different and no designation

CROSS-SECTIONAL STUDIES

Note: A study can be awarded a maximum of one star for Selection questions 1-3 and Outcome question 3. A maximum of two stars can be given for Selection question 4, Comparability question 1 and Outcome question 1. Overall maximum points is 10 for any individual study.

Selection: (Maximum 5 stars)

1) Representativeness of the sample
   a) consecutive or obviously representative series of cases *
   b) Selected group of users.
   c) potential for selection biases or not stated

2) Sample size:
   a) justified or satisfactory (≥100 participants). *
   b) not justified.

3) Non-respondents:
   a) same rate for both groups *
   b) non respondents described
   c) rate different and no designation

4) Ascertainment of the exposure (risk factor):
   a) uses validated measurement tool (i.e. KDIGO, AKIN or RIFLE criteria). **
   b) does not use independently validated criteria.
   c) no description

Comparability: (Maximum 2 stars)

1) Comparability of cases and controls on the basis of the design or analysis
   a) study controls for baseline demographics (age/sex). *
   b) study controls for comorbidities (i.e. DM, HTN, CKD). *
   c) study not controlled for relevant confounders or information not provided.

Outcome: (Maximum 3 stars)

1) Assessment of the outcome:
   a) Objective validated laboratory method (i.e. qRT-PCR/microarray/sequencing). **
   b) Uses non-standard or non-validated laboratory methods.
   c) No description/non-standard laboratory methods used.

2) Statistical test:
   a) The statistical test used is clearly described and appropriate, and the measurement of the association is presented, including confidence intervals and the probability level (p value). *
   b) The statistical test is not appropriate, not described or incomplete.
