# Supplementary material for: A Systematic Review and Meta-Analysis of MicroRNA as Predictive Biomarkers of Acute Kidney Injury
Source: Biomedicines. 2024 Jul 30;12(8):1695. doi: 10.3390/biomedicines12081695 (PMC11351452; doi:10.3390/biomedicines12081695)
Supplement: Supplementary file 1 [file biomedicines-12-01695-s001.zip › Table S1.pdf]

Table S1: Online databases and searched terms used to identify systematic review studies

Database	Search Criteria	n
PubMed	(ischaemia OR ischemia OR ischaemic OR ischemic OR ischaemia reperfusion OR ischemia reperfusion OR shock OR trauma OR sepsis OR nephrotoxicity OR radiocontrast OR Cardiopulmonary bypass OR cardiac surgery) AND (kidney OR renal OR kidney injury OR renal injury) AND (miRNA OR mi-RNA OR microRNA OR micro-RNA OR miR OR mi-R) AND human NOT cancer	595
Cochrane Library	(ischaemia OR ischemia OR ischaemic OR ischemic OR ischaemia reperfusion OR ischemia reperfusion OR shock OR trauma OR sepsis OR nephrotoxicity OR radiocontrast OR Cardiopulmonary bypass OR cardiac surgery) AND (kidney OR renal OR kidney injury OR renal injury) AND (miRNA OR mi-RNA OR microRNA OR micro-RNA OR miR OR mi-R) AND human NOT cancer	25
Scopus	TITLE-ABS-KEY ((ischaemia OR ischemia OR ischaemic OR ischemic OR "ischaemia reperfusion" OR "ischemia reperfusion" OR shock OR trauma OR sepsis OR nephrotoxicity OR radiocontrast OR "Cardiopulmonary bypass" OR "cardiac surgery") AND (kidney OR renal OR "kidney injury" OR "renal injury") AND (mirna OR mi-rna OR microrna OR micro-rna OR mir OR mi-r) AND human AND NOT cancer)	816
Ovid Medline	(((ischaemia or ischemia or ischaemic or ischemic or ischaemia reperfusion or ischemia reperfusion or shock or trauma or sepsis or nephrotoxicity or radiocontrast or Cardiopulmonary bypass or cardiac surgery) and (kidney or renal or kidney injury or renal injury) and (miRNA or mi-RNA or microRNA or micro-RNA or miR or mi-R) and human) not cancer).mp.	262
