# Supplementary material for: A Systematic Review and Meta-Analysis of MicroRNA as Predictive Biomarkers of Acute Kidney Injury
Source: Biomedicines. 2024 Jul 30;12(8):1695. doi: 10.3390/biomedicines12081695 (PMC11351452; doi:10.3390/biomedicines12081695)
Supplement: Supplementary file 1 [file biomedicines-12-01695-s001.zip › Table S2.pdf]

Table S2: Summary of included study characteristics

Title	Authors	Year	DOI	Cohort	Clinical characteristics	AKI assessment criteria	Sample used	Cohort size	Age Category
A Pilot Study Identifying a Set of microRNAs As Precise Diagnostic Biomarkers of Acute Kidney Injury	Aguado-Fraile et al.	2015	10.1371/journal.pone.0127175	Cross-sectional	unknown, ICU	AKIN	Serum	39	Adult
				Case-control	Cardiovascular surgery	AKIN, RIFLE, SCr	Serum	41	Adult
MicroRNA expression profiling in acute kidney injury	Aomatsu et al.	2022	10.1016/j.trsl.2021.11.010	Cross-sectional	ICU	KDIGO	Serum	30	Adult
Early detection of cardiac surgery-associated acute kidney injury by microRNA-21	Arvin et al.	2017	10.4149/bll_2017_120	Case-control	Cardiovascular surgery	KDIGO	Serum, Urine	28	Adult
Urinary miR-16 transactivated by C/EBPβ reduces kidney function after ischemia/reperfusion–induced injury	Chen et al.	2016	10.1038/srep27945	Cross-sectional	ICU	AKIN, RIFLE	Serum, Urine	18	Adult

Circ_0001806 relieves LPS-induced HK2 cell injury by regulating the expression of miR-942-5p and TXNIP	Chen et al.	2023	10.1007/s10863-023-09978-3	Cross-sectional	Sepsis	Unknown	Serum	80	Adult
MicroRNA-21 and Risk of Severe Acute Kidney Injury and Poor Outcomes after Adult Cardiac Surgery	Du et al.	2013	10.1371/journal.pone.0063390	Case-control	Cardiovascular surgery	AKIN	Urine, Plasma	120	Adult
A circulating miRNA signature for early diagnosis of acute kidney injury following acute myocardial infarction	Fan et al.	2019	10.1186/s12967-019-1890-7	Cross-sectional	ICU	KDIGO	Serum	59	Adult
NEAT1 aggravates sepsis-induced acute kidney injury by sponging miR-22-3p	Feng et al.	2020	10.1515/med-2020-0401	Cross-sectional	Sepsis	Unknown	Serum	18	Unspecified
circSTRN3 aggravates sepsis-induced	Gao et al.	2022	10.1080/21655979.2022.2061293	Cross-sectional	Sepsis	Unknown	Serum	30	Unspecified

acute kidney injury by regulating miR-578/ toll like receptor 4 axis									
Differentially expressed miRNAs in sepsis-induced acute kidney injury target oxidative stress and mitochondrial dysfunction pathways	Ge et al.	2017	10.1371/journal.pone.0173292	Cross-sectional	Sepsis	KDIGO	Serum	65	Adult
Hsa-miR-494-3p attenuates gene HtrA3 transcription to increase inflammatory response in hypoxia/reoxygenation HK2 Cells	Gong et al.	2021	10.1038/s41598-021-81113-x	Case-control	Cardiovascular surgery	KDIGO	Serum	18	Adult
Downregulation of circ-ZNF644 alleviates LPS-induced HK2 cell injury via miR-335-5p/HIPK1 axis	Gong et al.	2022	10.1002/tox.23642	Cross-sectional	Sepsis	AKIN	Serum	38	Adult

Dysregulated microRNAs involved in contrast-induced acute kidney injury in rat and human	Gutiérrez-Escolano et al.	2015	10.3109/0886022X.2015.1077322	Case-control	Nephrotoxicity (contrast)	SCr	Plasma	184	Adult
Mechanism of circHIPK3-miRNA-124-3p/miRNA-148b-3p-Mediated Inflammatory Responses and Cell Senescence in Candida albicans-Induced Septic Acute Kidney Injury	Han et al.	2022	10.1159/000523910	Cross-sectional	Sepsis	KDIGO	Serum	30	Adult
LncRNA NEAT1 promotes hypoxia-induced renal tubular epithelial apoptosis through downregulating miR-27a-3p	Jiang et al.	2019	10.1002/jcb.28909	Cross-sectional	Ischemia	Unknown	Serum	18	Unspecified
CIRC_0001818 TARGETS MIR-136-5P TO INCREASE	Kuang et al.	2023	10.1097/SHK.0000000000002140	Cross-sectional	Sepsis	Unknown	Serum	25	Adult

LIPOPOLYSACCHARIDE-INDUCED HK2 CELL INJURIES BY ACTIVATING TXNIP/NLRP3 INFLAMMASOME PATHWAY									
MicroRNA-494 Reduces ATF3 Expression and Promotes AKI	Lan et al.	2012	10.1681/ASN.2012050438	Cross-sectional	ICU	AKIN	Serum, Urine	26	Adult
Knockdown of circ-FANCA alleviates LPS-induced HK2 cell injury via targeting miR-93-5p/OXSR1 axis in septic acute kidney injury	Li et al.	2021	10.1186/s13098-021-00625-8	Cross-sectional	Sepsis	Unknown	Serum	19	Adult
Circ_0040994 depletion alleviates lipopolysaccharide-induced HK2 cell injury through miR-17-5p/TRPM7 axis	Li et al.	2023	10.1002/tox.23894	Cross-sectional	Sepsis	Unknown	Serum	30	Adult
Inhibition of MiR-106b-5p mediated by exosomes mitigates acute	Li et al.	2023a	10.1111/jcmm.17848	Case-control	Cardiovascular surgery	KDIGO	Urine	66	Adult

kidney injury by modulating transmissible endoplasmic reticulum stress and M1 macrophage polarization									
Inhibition of sepsis-induced acute kidney injury via the circITCH-miR-579-3p-ZEB2 axis	Li et al.	2023b	10.1002/tox.23682	Cross-sectional	Sepsis	Unknown	Serum	20	Adult
CircNRIP1 KNOCKDOWN ALLEVIATES LIPOPOLYSACCHARIDE-INDUCED HUMAN KIDNEY 2 CELL APOPTOSIS AND INFLAMMATION THROUGH miR-339-5p/OXSR1 PATHWAY	Li et al.	2023c	10.1097/SHK.0000000000002057	Cross-sectional	Sepsis	Unknown	Serum	25	Adult
Expression patterns and prognostic value of miR-210, miR-494, and miR-205 in	Lin et al.	2019	10.17305/bjbms.2019.4131	Cross-sectional	Sepsis	AKIN	Serum	110	Adult

middle-aged and old patients with sepsis-induced acute kidney injury									
Discovery and validation of miR-452 as an effective biomarker for acute kidney injury in sepsis	Liu et al.	2020	10.7150/thno.50093	Cross-sectional	Sepsis	KDIGO	Serum, Urine	97	Adult
The negative feedback loop of NF-κB/miR-376b/NFKBIZ in septic acute kidney injury	Liu et al.	2020a	10.1172/jci.insight.142272	Cross-sectional	Sepsis	KDIGO	Serum, Urine	40	Adult
Downregulation of miR-574-5p inhibits HK-2 cell viability and predicts the onset of acute kidney injury in sepsis patients	Liu et al.	2021	10.1080/0886022X.2021.1939051	Cross-sectional	Sepsis	KDIGO, AKIN	Serum	136	Adult
Exosomal microRNA-342-5p secreted from adipose-derived mesenchymal stem cells mitigates acute	Liu et al.	2023	10.1186/s12575-023-00198-y	Cross-sectional	Sepsis	KDIGO	Serum	30	Adult

kidney injury in sepsis mice by inhibiting TLR9									
Circulating miR-210 Predicts Survival in Critically Ill Patients with Acute Kidney Injury	Lorenzen et al.	2011	10.2215/CJN.00430111	Cross-sectional	Unknown	RIFLE	Plasma	82	Adult
SIKIAT1/miR-96/FOXA1 axis regulates sepsis-induced kidney injury through induction of apoptosis	Lu et al.	2020	10.1007/s00011-020-01350-0	Cross-sectional	Sepsis	Unknown	PBMC	10	Unspecified
Circular RNA HIPK3 aggravates sepsis-induced acute kidney injury via modulating the microRNA-338/forkhead box A1 axis	Lu et al.	2022	10.1080/21655979.2022.2032974	Cross-sectional	Sepsis	Unknown	Serum	23	Unspecified
A novel role of the miR-152-3p/ERRFI1/STAT3 pathway modulates the apoptosis and inflammatory	Ma et al.	2020	10.1002/jbt.22540	Cross-sectional	Sepsis	Unknown	Serum	21	Adult

response after acute kidney injury									
The Potential of miR-370-3p and miR-495-3p Serving as Biomarkers for Sepsis-Associated Acute Kidney Injury	Ma et al.	2022	10.1155/2022/2439509	Cross-sectional	Sepsis	KDIGO, AKIN	Urine	88	Adult
MEG3 aggravates hypoxia/reoxygenation induced apoptosis of renal tubular epithelial cells via the miR-129-5p/HMGB1 axis	Mao et al.	2021	10.1002/jbt.22649	Cross-sectional	Unknown	Unknown	Serum	25	Unspecified
Urinary extracellular vesicles and micro-RNA as markers of acute kidney injury after cardiac surgery	Miller et al.	2022	10.1038/s41598-022-13849-z	Case-control	Cardiovascular surgery	KDIGO	Urine	94	Adult
miR-141 mediates recovery from	Newbury et al.	2021	10.1038/s41598-021-94984-x	Cross-sectional	Unknown	KDIGO	Urine	29	Adult

acute kidney injury									
Detection of Drug-Induced Acute Kidney Injury in Humans Using Urinary KIM-1, miR-21, -200c, and -423	Pavkovic et al.	2016	10.1093/toxsci/kfw077	Cross-sectional	Nephrotoxicity (APAP)	SCr	Urine	70	Adult
Human miRNome Profiling Identifies MicroRNAs Differentially Present in the Urine after Kidney Injury	Ramachandran et al.	2013	10.1373/clinchem.2013.210245	Cross-sectional	ICU	KDIGO	Urine	94	Adult
Expression, Circulation, and Excretion Profile of MicroRNA-21, -155, and -18a Following Acute Kidney Injury	Saikumar et al.	2012	10.1093/toxsci/kfs210	Cross-sectional	Unknown	Unknown	Urine	22	Adult
MiR-150-5p protects against septic acute kidney injury via repressing the MEKK3/JNK pathway	Shi et al.	2021	10.1016/j.cellsig.2021.110101	Cross-sectional	Sepsis	KDIGO	Serum	30	Adult

Urinary versus serum microRNAs in human oxalic acid poisoning: Contrasting signals and performance	Shihana et al.	2020	10.1016/j.toxlet.2020.09.003	Case-control	Nephrotoxicity (oxalic acid)	AKIN	Serum, Urine	84	Adult
An Observational Cohort Feasibility Study to Identify Microvesicle and Micro-RNA Biomarkers of Acute Kidney Injury Following Pediatric Cardiac Surgery	Sullo et al.	2018	10.1097/PCC.0000000000001604	Case-control	Cardiovascular surgery	KDIGO	Serum	48	Paediatric
Circulating MicroRNA-188, -30a, and -30e as Early Biomarkers for Contrast-Induced Acute Kidney Injury	Sun et al.	2016	10.1161/JAHA.116.004138	Case-control	Nephrotoxicity (contrast)	SCr	Plasma	142	Adult
Correlation Between Single Nucleotide Polymorphisms at the 3′-UTR	Sun et al.	2020	10.1089/gtmb.2019.0222	Cross-sectional	Sepsis	SCr	Plasma	470	Adult

of the NFKB1 Gene and Acute Kidney Injury in Sepsis									
Circ_0091702 serves as a sponge of miR-545-3p to attenuate sepsis-related acute kidney injury by upregulating THBS2	Tan et al.	2021	10.1007/s10735-021-09991-z	Cross-sectional	Sepsis	Unknown	Serum	33	Unspecified
Downregulation of XIST ameliorates acute kidney injury by sponging miR-142-5p and targeting PDCD4	Tang et al.	2020	10.1002/jcp.29729	Cross-sectional	Unknown	Unknown	Serum	100	Unspecified
MiR-107 induces TNF-α secretion in endothelial cells causing tubular cell injury in patients with septic acute kidney injury	Wang et al.	2017	10.1016/j.bbrc.2017.01.013	Cross-sectional	Unknown	KDIGO	Circulating endothelial cells	30	Paediatric
Down-regulation of	Wang et al.	2021	10.1093/jb/mvab008	Cross-sectional	Sepsis	Unknown	Serum	105	Adult

lncRNA SNHG5 relieves sepsis-induced acute kidney injury by regulating the miR-374a-3p/TLR4/NF-κB pathway									
Silencing circ_0074371 inhibits the progression of sepsis-induced acute kidney injury by regulating miR-330-5p/ELK1 axis	Wang et al.	2022	10.1007/s00335-022-09961-0	Cross-sectional	Sepsis	Unknown	Serum	30	Unspecified
microRNA-338-3p suppresses lipopolysaccharide-induced inflammatory response in HK-2 cells	Wang et al.	2022a	10.1186/s12860-022-00455-0	Cross-sectional	Sepsis	Unknown	Serum	Unspecified	Unspecified
CircVMA21 ameliorates lipopolysaccharide (LPS)-induced HK-2 cell injury depending on the regulation of miR-7-5p/PPARA	Wang et al.	2022b	10.1080/08916934.2021.2012764	Cross-sectional	Sepsis	Unknown	Serum	41	Unspecified

MicroRNA-668 represses MTP18 to preserve mitochondrial dynamics in ischemic acute kidney injury	Wei et al.	2018	10.1172/JCI121859	Cross-sectional	Unknown	SCr	Serum, Urine, Kidney Biopsy	8	Adult
				Case-control	Cardiovascular surgery	SCr	Serum, Urine	62	Adult
The miR-15a-5p-XIST-CUL3 regulatory axis is important for sepsis-induced acute kidney injury	Xu et al.	2019	10.1080/0886022X.2019.1669460	Cross-sectional	Unknown	Unknown	Blood RNA	5	Adult
miR-195-5p alleviates acute kidney injury through repression of inflammation and oxidative stress by targeting vascular endothelial growth factor A	Xu et al.	2020	10.18632/aging.103160	Cross-sectional	Unknown	Unknown	Serum	80	Unspecified
Circ_0114427 promotes LPS-induced septic acute kidney injury by modulating miR-495-	Xu et al.	2022	10.1080/08916934.2021.1995861	Cross-sectional	Sepsis	Unknown	Serum	42	Adult

3p/TRAF6 through the NF-κB pathway									
THE VALUE OF COMBINING MIR-10A-5P LEVELS AND PLR TO EVALUATE THE PROGNOSIS OF SEPSIS PATIENTS WITH ACUTE KIDNEY INJURY	Xun et al.	2022	10.19193/0393-6384_2022_5_487	Cross-sectional	Sepsis	Unknown	Serum	142	Adult
Long non-coding RNA SNHG14 aggravates LPS-induced acute kidney injury through regulating miR-495-3p/HIPK1	Yang et al.	2021	10.1093/abbs/gmab034	Cross-sectional	Sepsis	Unknown	Serum	20	Unspecified
miR-23a-3p inhibits sepsis-induced kidney epithelial cell injury by suppressing Wnt/β-catenin signaling by targeting wnt5a	Ye et al.	2022	10.1590/1414-431X2021e11571	Cross-sectional	Sepsis	AKIN	Serum	25	Adult
CIRC_0008882 STIMULATES PDE7A TO	You et al.	2023	10.1097/SHK.0000000000002093	Cross-sectional	Sepsis	Unknown	Serum	36	Adult

SUPPRESS SEPTIC ACUTE KIDNEY INJURY PROGRESSION BY SPONGING MIR-155-5P									
LncRNA PVT1 accelerates LPS-induced septic acute kidney injury through targeting miR-17-5p and regulating NF-κB pathway	Yuan et al.	2021	10.1007/s11255-021-02905-8	Cross-sectional	Sepsis	KDIGO	Serum	25	Adult
Implications of dynamic changes in miR-192 expression in ischemic acute kidney injury	Zhang et al.	2017	10.1007/s11255-016-1485-7	Case-control	Cardiovascular surgery	KDIGO	Plasma	70	Adult
Urinary miR-26b as a potential biomarker for patients with sepsis-associated acute kidney injury: a Chinese population-based study	Zhang et al.	2018	10.26355/eurrev_201807_15518	Cross-sectional	Sepsis	KDIGO, AKIN	Urine	155	Adult

Long Non-Coding RNA RMRP Contributes to Sepsis-Induced Acute Kidney Injury	Zhang et al.	2021	10.3349/ymj.2021.62.3.262	Cross-sectional	Sepsis	Unknown	Serum	48	Unspecified
Deregulated microRNA-22-3p in patients with sepsis-induced acute kidney injury serves as a new biomarker to predict disease occurrence and 28-day survival outcomes	Zhang et al.	2021a	10.1007/s11255-021-02784-z	Cross-sectional	Sepsis	KDIGO, AKIN	Serum, Urine	158	Adult
CIRC_0002131 CONTRIBUTES TO LPS-INDUCED APOPTOSIS, INFLAMMATION, AND OXIDATIVE INJURY IN HK-2 CELLS VIA INHIBITING THE BINDING BETWEEN MIR-942-5P AND OXSR1	Zhang et al.	2023	10.1097/SHK.0000000000002197	Cross-sectional	Sepsis	Unknown	Serum	35	Adult

LncRNA PMS2L2 Is Downregulated in Sepsis-Induced Acute Kidney Injury and Inhibits LPS-Induced Apoptosis of Podocytes	Zhang et al,	2023a	10.1159/000528053	Cross-sectional	Sepsis	SCr	Serum	100	Adult
CIRC_0114428 INFLUENCES THE PROGRESSION OF SEPTIC ACUTE KIDNEY INJURY VIA RE GULATING MIR-370-3P/TIMP2 AXIS	Zhang et al.	2023b	10.1097/SHK.0000000000002077	Cross-sectional	Sepsis	Unknown	Serum	45	Adult
Long Noncoding RNA DANCR Suppressed Lipopolysaccharide-Induced Septic Acute Kidney Injury by Regulating miR-214 in HK-2 Cells	Zhao et al.	2020	10.12659/MSM.921822	Cross-sectional	Sepsis	AKIN	Serum	20	Adult
miR-34b-5p promotes renal cell inflammation and apoptosis	Zheng et al.	2021	10.1080/0886022X.2021.1871922	Cross-sectional	Sepsis	KDIGO	Serum	30	Adult

by inhibiting aquaporin-2 in sepsis-induced acute kidney injury									
Circ-BNIP3L knockdown alleviates LPS-induced renal tubular epithelial cell injury during sepsis-associated acute kidney injury by miR-370-3p/MYD88 axis	Zhou et al.	2021	10.1007/s10863-021-09925-0	Cross-sectional	Sepsis	AKIN	Plasma	17	Unspecified
Circ_0006944 aggravates LPS-induced HK2 cell injury via modulating miR-205-5p/UBL4A pathway	Zhou et al.	2023	10.1080/08916934.2023.2276066	Cross-sectional	Sepsis	Unknown	Serum	19	Unspecified
Urinary MicroRNA-30c-5p and MicroRNA-192-5p as potential biomarkers of ischemia–reperfusion-induced kidney injury	Zou et al.	2017	10.1177/15353702166850	Case-control	Cardiovascular surgery	KDIGO	Urine	71	Adult

1. Aguado-Fraile, E.; Ramos, E.; Conde, E.; Rodríguez, M.; Martín-Gómez, L.; Lietor, A.; Candela, Á.; Ponte, B.; Liaño, F.; García-Bermejo, M.L. A Pilot Study Identifying a Set of microRNAs As Precise Diagnostic Biomarkers of Acute Kidney Injury. *PLoS ONE* **2015**, *10*, e0127175, doi:10.1371/journal.pone.0127175.
2. Aomatsu, A.; Kaneko, S.; Yanai, K.; Ishii, H.; Ito, K.; Hirai, K.; Ookawara, S.; Kobayashi, Y.; Sanui, M.; Morishita, Y. MicroRNA expression profiling in acute kidney injury. *Transl Res* **2022**, *244*, 1-31, doi:https://dx.doi.org/10.1016/j.trsl.2021.11.010.
3. Arvin, P.; Samimagham, H.R.; Montazerghaem, H.; Khayatian, M.; Mahboobi, H.; Ghadiri Soufi, F. Early detection of cardiac surgery-associated acute kidney injury by microRNA-21. *Bratisl Lek Listy* **2017**, *118*, 626-631, doi:https://dx.doi.org/10.4149/BLL_2017_120.
4. Chen, H.H.; Lan, Y.F.; Li, H.F.; Cheng, C.F.; Lai, P.F.; Li, W.H.; Lin, H. Urinary miR-16 transactivated by C/EBPβ reduces kidney function after ischemia/reperfusion-induced injury. *Scientific reports* **2016**, *6*, 27945, doi:10.1038/srep27945.
5. Chen, M.; Zhang, L. Circ_0001806 relieves LPS-induced HK2 cell injury by regulating the expression of miR-942-5p and TXNIP. *J Bioenerg Biomembr* **2023**, *55*, 301-312, doi:https://dx.doi.org/10.1007/s10863-023-09978-3.
6. Du, J.; Cao, X.; Zou, L.; Chen, Y.; Guo, J.; Chen, Z.; Hu, S.; Zheng, Z. MicroRNA-21 and risk of severe acute kidney injury and poor outcomes after adult cardiac surgery. *PLoS ONE* **2013**, *8*, e63390, doi:https://dx.doi.org/10.1371/journal.pone.0063390.
7. Fan, P.C.; Chen, C.C.; Peng, C.C.; Chang, C.H.; Yang, C.H.; Yang, C.; Chu, L.J.; Chen, Y.C.; Yang, C.W.; Chang, Y.S.; Chu, P.H. A circulating miRNA signature for early diagnosis of acute kidney injury following acute myocardial infarction. *J Transl Med* **2019**, *17*, 139, doi:10.1186/s12967-019-1890-7.
8. Feng, Y.; Liu, J.; Wu, R.; Yang, P.; Ye, Z.; Song, F. NEAT1 aggravates sepsis-induced acute kidney injury by sponging miR-22-3p. **2020**, *15*, 333-342, doi:doi:10.1515/med-2020-0401.
9. Gao, Q.; Zheng, Y.; Wang, H.; Hou, L.; Hu, X. circSTRN3 aggravates sepsis-induced acute kidney injury by regulating miR-578/ toll like receptor 4 axis. *Bioengineered* **2022**, *13*, 11388-11401, doi:10.1080/21655979.2022.2061293.
10. Ge, Q.M.; Huang, C.M.; Zhu, X.Y.; Bian, F.; Pan, S.M. Differentially expressed miRNAs in sepsis-induced acute kidney injury target oxidative stress and mitochondrial dysfunction pathways. *PLoS ONE* **2017**, *12*, e0173292, doi:10.1371/journal.pone.0173292.
11. Gong, J.; Zhao, S.; Luo, S.; Yin, S.; Li, X.; Feng, Y. Downregulation of circ-ZNF644 alleviates LPS-induced HK2 cell injury via miR-335-5p/HIPK1 axis. *Environ Toxicol* **2022**, *37*, 2855-2864, doi:https://dx.doi.org/10.1002/tox.23642.
12. Gong, Q.; Shen, Z.-M.; Sheng, Z.; Jiang, S.; Ge, S.-L. Hsa-miR-494-3p attenuates gene HtrA3 transcription to increase inflammatory response in hypoxia/reoxygenation HK2 Cells. *Scientific reports* **2021**, *11*, 1665, doi:https://dx.doi.org/10.1038/s41598-021-81113-x.
13. Gutiérrez-Escolano, A.; Santacruz-Vázquez, E.; Gómez-Pérez, F. Dysregulated microRNAs involved in contrast-induced acute kidney injury in rat and human. *Renal Failure* **2015**, *37*, 1498-1506, doi:10.3109/0886022X.2015.1077322.
14. Han, J.; Li, W.; Zhang, J.; Guan, Y.; Huang, Y.; Li, X. Mechanism of circHIPK3-miRNA-124-3p/miRNA-148b-3p-Mediated Inflammatory Responses and Cell Senescence in Candida albicans -Induced Septic Acute Kidney Injury. *Gerontology* **2022**, *68*, 1145-1165, doi:10.1159/000523910.
15. Jiang, X.; Li, D.; Shen, W.; Shen, X.; Liu, Y. LncRNA NEAT1 promotes hypoxia-induced renal tubular epithelial apoptosis through downregulating miR-27a-3p. *J Cell Biochem* **2019**, *120*, 16273-16282, doi:https://dx.doi.org/10.1002/jcb.28909.

16. Kuang, F.; Wang, B.; You, T.; Liu, Y.; Li, P.; Wang, J.; Peng, L. CIRC_0001818 TARGETS MIR-136-5P TO INCREASE LIPOPOLYSACCHARIDE-INDUCED HK2 CELL INJURIES BY ACTIVATING TXNIP/NLRP3 INFLAMMASOME PATHWAY. *Shock* **2023**, *60*, 110-120, doi:https://dx.doi.org/10.1097/SHK.0000000000002140.
17. Lan, Y.-F.; Chen, H.-H.; Lai, P.-F.; Cheng, C.-F.; Huang, Y.-T.; Lee, Y.-C.; Chen, T.-W.; Lin, H. MicroRNA-494 reduces ATF3 expression and promotes AKI. *J Am Soc Nephrol* **2012**, *23*, 2012-2023, doi:https://dx.doi.org/10.1681/ASN.2012050438.
18. Li, H.; Zhang, X.; Wang, P.; Zhou, X.; Liang, H.; Li, C. Knockdown of circ-FANCA alleviates LPS-induced HK2 cell injury via targeting miR-93-5p/OXSR1 axis in septic acute kidney injury. *Diabetology and Metabolic Syndrome* **2021**, *13*, doi:10.1186/s13098-021-00625-8.
19. Li, P.; Liu, Y.; You, T. CircNRIP1 KNOCKDOWN ALLEVIATES LIPOPOLYSACCHARIDE-INDUCED HUMAN KIDNEY 2 CELL APOPTOSIS AND INFLAMMATION THROUGH miR-339-5p/OXSR1 PATHWAY. *Shock* **2023**, *59*, 426-433, doi:https://dx.doi.org/10.1097/SHK.0000000000002057.
20. Li, Q.; Wang, T.; Wang, X.; Ge, X.-Y.; Yang, T.; Bai, G.; Wang, W. Inhibition of sepsis-induced acute kidney injury via the circITCH-miR-579-3p-ZEB2 axis. *Environ Toxicol* **2023**, *38*, 1217-1225, doi:https://dx.doi.org/10.1002/tox.23682.
21. Li, X.; Zhong, Y.; Yue, R.; Xie, J.; Zhang, Y.; Lin, Y.; Li, H.; Xu, Y.; Zheng, D. Inhibition of MiR-106b-5p mediated by exosomes mitigates acute kidney injury by modulating transmissible endoplasmic reticulum stress and M1 macrophage polarization. *Journal of Cellular and Molecular Medicine* **2023**, *27*, 2876-2889, doi:10.1111/jcmm.17848.
22. Li, Y.; Chai, Y. Circ_0040994 depletion alleviates lipopolysaccharide-induced HK2 cell injury through miR-17-5p/TRPM7 axis. *Environ Toxicol* **2023**, *38*, 2585-2594, doi:https://dx.doi.org/10.1002/tox.23894.
23. Lin, Y.; Ding, Y.; Song, S.; Li, M.; Wang, T.; Guo, F. Expression patterns and prognostic value of miR-210, miR-494, and miR-205 in middle-aged and old patients with sepsis-induced acute kidney injury. *Bosn. j. basic med. sci.* **2019**, *19*, 249-256, doi:https://dx.doi.org/10.17305/bjbms.2019.4131.
24. Liu, S.; Zhao, L.; Zhang, L.; Qiao, L.; Gao, S. Downregulation of miR-574-5p inhibits HK-2 cell viability and predicts the onset of acute kidney injury in sepsis patients. *Renal failure* **2021**, *43*, 942-948, doi:https://dx.doi.org/10.1080/0886022X.2021.1939051.
25. Liu, W.; Hu, C.; Zhang, B.; Li, M.; Deng, F.; Zhao, S. Exosomal microRNA-342-5p secreted from adipose-derived mesenchymal stem cells mitigates acute kidney injury in sepsis mice by inhibiting TLR9. *Biol. proced. online* **2023**, *25*, 10, doi:https://dx.doi.org/10.1186/s12575-023-00198-y.
26. Liu, Z.; Tang, C.; He, L.; Yang, D.; Cai, J.; Zhu, J.; Shu, S.; Liu, Y.; Yin, L.; Chen, G.; et al. The negative feedback loop of NF-kappaB/miR-376b/NFKBIZ in septic acute kidney injury. *JCI insight* **2020**, *5*, doi:https://dx.doi.org/10.1172/jci.insight.142272.
27. Liu, Z.; Yang, D.; Gao, J.; Xiang, X.; Hu, X.; Li, S.; Wu, W.; Cai, J.; Tang, C.; Zhang, D.; Dong, Z. Discovery and validation of miR-452 as an effective biomarker for acute kidney injury in sepsis. *Theranostics* **2020**, *10*, 11963-11975, doi:https://dx.doi.org/10.7150/thno.50093.
28. Lorenzen, J.M.; Kielstein, J.T.; Hafer, C.; Gupta, S.K.; Kümpers, P.; Faulhaber-Walter, R.; Haller, H.; Fliser, D.; Thum, T. Circulating miR-210 Predicts Survival in Critically Ill Patients with Acute Kidney Injury. *Clinical Journal of the American Society of Nephrology* **2011**, *6*.
29. Lu, H.; Chen, Y.; Wang, X.; Yang, Y.; Ding, M.; Qiu, F. Circular RNA HIPK3 aggravates sepsis-induced acute kidney injury via modulating the microRNA-338/forkhead box A1 axis. *Bioengineered* **2022**, *13*, 4798-4809, doi:https://dx.doi.org/10.1080/21655979.2022.2032974.
30. Lu, S.; Wu, H.; Xu, J.; He, Z.; Li, H.; Ning, C. SIKIAT1/miR-96/FOXA1 axis regulates sepsis-induced kidney injury through induction of apoptosis. *Inflamm Res* **2020**, *69*, 645-656, doi:https://dx.doi.org/10.1007/s00011-020-01350-0.

31. Ma, P.; Zhang, C.; Huo, P.; Li, Y.; Yang, H. A novel role of the miR-152-3p/ERRFI1/STAT3 pathway modulates the apoptosis and inflammatory response after acute kidney injury. *Journal of Biochemical and Molecular Toxicology* **2020**, *34*, doi:10.1002/jbt.22540.
32. Ma, W.; Miao, X.; Xia, F.; Ruan, C.; Tao, D.; Li, B. The Potential of miR-370-3p and miR-495-3p Serving as Biomarkers for Sepsis-Associated Acute Kidney Injury. *Comput. math. methods med.* **2022**, *2022*, 2439509, doi:https://dx.doi.org/10.1155/2022/2439509.
33. Mao, H.; Huang, Q.; Liu, Y. MEG3 aggravates hypoxia/reoxygenation induced apoptosis of renal tubular epithelial cells via the miR-129-5p/HMGB1 axis. *Journal of biochemical and molecular toxicology* **2021**, *35*, e22649, doi:https://dx.doi.org/10.1002/jbt.22649.
34. Miller, D.; Eagle-Hemming, B.; Sheikh, S.; Joel-David, L.; Adebayo, A.; Lai, F.Y.; Roman, M.; Kumar, T.; Aujla, H.; Murphy, G.J.; Woźniak, M.J. Urinary extracellular vesicles and micro-RNA as markers of acute kidney injury after cardiac surgery. *Scientific reports* **2022**, *12*, 10402, doi:10.1038/s41598-022-13849-z.
35. Newbury, L.J.; Simpson, K.; Khalid, U.; John, I.; de Rivera, L.B.; Lu, Y.-A.; Lopez-Anton, M.; Watkins, W.J.; Jenkins, R.H.; Fraser, D.J.; Bowen, T. miR-141 mediates recovery from acute kidney injury. *Scientific reports* **2021**, *11*, 16499, doi:https://dx.doi.org/10.1038/s41598-021-94984-x.
36. Pavkovic, M.; Robinson-Cohen, C.; Chua, A.S.; Nicoara, O.; Cardenas-Gonzalez, M.; Bijol, V.; Ramachandran, K.; Hampson, L.; Pirmohamed, M.; Antoine, D.J.; et al. Detection of Drug-Induced Acute Kidney Injury in Humans Using Urinary KIM-1, miR-21, -200c, and -423. *Toxicol Sci* **2016**, *152*, 205-213, doi:https://dx.doi.org/10.1093/toxsci/kfw077.
37. Ramachandran, K.; Saikumar, J.; Bijol, V.; Koyner, J.L.; Qian, J.; Betensky, R.A.; Waikar, S.S.; Vaidya, V.S. Human miRNome Profiling Identifies MicroRNAs Differentially Present in the Urine after Kidney Injury. *Clinical Chemistry* **2013**, *59*, 1742-1752, doi:10.1373/clinchem.2013.210245.
38. Saikumar, J.; Hoffmann, D.; Kim, T.-M.; Gonzalez, V.R.; Zhang, Q.; Goering, P.L.; Brown, R.P.; Bijol, V.; Park, P.J.; Waikar, S.S.; Vaidya, V.S. Expression, circulation, and excretion profile of microRNA-21, -155, and -18a following acute kidney injury. *Toxicol Sci* **2012**, *129*, 256-267, doi:https://dx.doi.org/10.1093/toxsci/kfs210.
39. Shi, L.; Zhang, Y.; Xia, Y.; Li, C.; Song, Z.; Zhu, J. MiR-150-5p protects against septic acute kidney injury via repressing the MEKK3/JNK pathway. *Cell Signal* **2021**, *86*, 110101, doi:https://dx.doi.org/10.1016/j.cellsig.2021.110101.
40. Shihana, F.; Mohamed, F.; Joglekar, M.V.; Hardikar, A.A.; Seth, D.; Buckley, N.A. Urinary versus serum microRNAs in human oxalic acid poisoning: Contrasting signals and performance. *Toxicol Lett* **2020**, *334*, 21-26, doi:https://dx.doi.org/10.1016/j.toxlet.2020.09.003.
41. Sullo, N.; Mariani, S.; JnTala, M.; Kumar, T.; Woźniak, M.J.; Smallwood, D.; Pais, P.; Westrope, C.; Lotto, A.; Murphy, G.J. An Observational Cohort Feasibility Study to Identify Microvesicle and Micro-RNA Biomarkers of Acute Kidney Injury Following Pediatric Cardiac Surgery. *Pediatr Crit Care Med* **2018**, *19*, 816-830, doi:10.1097/PCC.0000000000001604.
42. Sun, J.; Cai, X.; Shen, J.; Jin, G.; Xie, Q. Correlation Between Single Nucleotide Polymorphisms at the 3'-UTR of the NFKB1 Gene and Acute Kidney Injury in Sepsis. *Genet Test Mol Biomarkers* **2020**, *24*, 274-284, doi:https://dx.doi.org/10.1089/gtmb.2019.0222.
43. Sun, S.q.; Zhang, T.; Ding, D.; Zhang, W.f.; Wang, X.l.; Sun, Z.; Hu, L.h.; Qin, S.y.; Shen, L.h.; He, B. Circulating Micro RNA-188,-30a, and-30e as Early Biomarkers for Contrast-Induced Acute Kidney Injury. *Journal of the American Heart Association* **2016**, *5*, e004138.
44. Tan, M.; Bei, R. Circ_0091702 serves as a sponge of miR-545-3p to attenuate sepsis-related acute kidney injury by upregulating THBS2. *J Mol Histol* **2021**, *52*, 717-728, doi:https://dx.doi.org/10.1007/s10735-021-09991-z.

45. Tang, B.; Li, W.; Ji, T.; Li, X.; Qu, X.; Feng, L.; Zhu, Y.; Qi, Y.; Zhu, C.; Bai, S. Downregulation of XIST ameliorates acute kidney injury by sponging miR-142-5p and targeting PDCD4. *J Cell Physiol* **2020**, *235*, 8852-8863, doi:https://dx.doi.org/10.1002/jcp.29729.
46. Wang, F.; Zhang, F.; Tian, Q.; Sheng, K. CircVMA21 ameliorates lipopolysaccharide (LPS)-induced HK-2 cell injury depending on the regulation of miR-7-5p/PPARA. *Autoimmunity* **2022**, *55*, 136-146, doi:https://dx.doi.org/10.1080/08916934.2021.2012764.
47. Wang, J.; Li, G.; Lin, M.; Lin, S.; Wu, L. microRNA-338-3p suppresses lipopolysaccharide-induced inflammatory response in HK-2 cells. *BMC Mol Cell Biol* **2022**, *23*, 60, doi:https://dx.doi.org/10.1186/s12860-022-00455-0.
48. Wang, M.; Wei, J.; Shang, F.; Zang, K.; Zhang, P. Down-regulation of lncRNA SNHG5 relieves sepsis-induced acute kidney injury by regulating the miR-374a-3p/TLR4/NF-κB pathway. *Journal of Biochemistry* **2021**, *169*, 575-583, doi:10.1093/jb/mvab008.
49. Wang, Q.-Y.; Zhang, R.-R.; Cui, L.; Sun, Y.-P. Silencing circ_0074371 inhibits the progression of sepsis-induced acute kidney injury by regulating miR-330-5p/ELK1 axis. *Mamm Genome* **2022**, *33*, 642-653, doi:https://dx.doi.org/10.1007/s00335-022-09961-0.
50. Wang, S.; Zhang, Z.; Wang, J.; Miao, H. MiR-107 induces TNF-alpha secretion in endothelial cells causing tubular cell injury in patients with septic acute kidney injury. *Biochem Biophys Res Commun* **2017**, *483*, 45-51, doi:https://dx.doi.org/10.1016/j.bbrc.2017.01.013.
51. Wei, Q.; Sun, H.; Song, S.; Liu, Y.; Liu, P.; Livingston, M.J.; Wang, J.; Liang, M.; Mi, Q.-S.; Huo, Y.; et al. MicroRNA-668 represses MTP18 to preserve mitochondrial dynamics in ischemic acute kidney injury. *J Clin Invest* **2018**, *128*, 5448-5464, doi:https://dx.doi.org/10.1172/JCI121859.
52. Xu, G.; Mo, L.; Wu, C.; Shen, X.; Dong, H.; Yu, L.; Pan, P.; Pan, K. The miR-15a-5p-XIST-CUL3 regulatory axis is important for sepsis-induced acute kidney injury. *Renal failure* **2019**, *41*, 955-966, doi:https://dx.doi.org/10.1080/0886022X.2019.1669460.
53. Xu, L.; Cao, H.; Xu, P.; Nie, M.; Zhao, C. Circ_0114427 promotes LPS-induced septic acute kidney injury by modulating miR-495-3p/TRAF6 through the NF-κB pathway. *Autoimmunity* **2022**, *55*, 52-64, doi:10.1080/08916934.2021.1995861.
54. Xu, Y.; Jiang, W.; Zhong, L.; Li, H.; Bai, L.; Chen, X.; Lin, Y.; Zheng, D. miR-195-5p alleviates acute kidney injury through repression of inflammation and oxidative stress by targeting vascular endothelial growth factor A. *Aging (Albany NY)* **2020**, *12*, 10235-10245, doi:https://dx.doi.org/10.18632/aging.103160.
55. Xun, L.; Li, Z.; Wang, H.; Zhang, P. THE VALUE OF COMBINING MIR-10A-5P LEVELS AND PLR TO EVALUATE THE PROGNOSIS OF SEPSIS PATIENTS WITH ACUTE KIDNEY INJURY. *Acta Medica Mediterranea* **2022**, *38*, 3303-3307, doi:10.19193/0393-6384_2022_5_488.
56. Yang, N.; Wang, H.; Zhang, L.; Lv, J.; Niu, Z.; Liu, J.; Zhang, Z. Long non-coding RNA SNHG14 aggravates LPS-induced acute kidney injury through regulating miR-495-3p/HIPK1. *Acta Biochim Biophys Sin (Shanghai)* **2021**, *53*, 719-728, doi:https://dx.doi.org/10.1093/abbs/gmab034.
57. Ye, J.; Feng, H.; Peng, Z. miR-23a-3p inhibits sepsis-induced kidney epithelial cell injury by suppressing Wnt/beta-catenin signaling by targeting wnt5a. *Braz J Med Biol Res* **2022**, *55*, e11571, doi:https://dx.doi.org/10.1590/1414-431X2021e11571.
58. You, T.; Kuang, F. CIRC_0008882 STIMULATES PDE7A TO SUPPRESS SEPTIC ACUTE KIDNEY INJURY PROGRESSION BY SPONGING MIR-155-5P. *Shock* **2023**, *59*, 657-665, doi:10.1097/SHK.0000000000002093.
59. Yuan, W.; Xiong, X.; Du, J.; Fan, Q.; Wang, R.; Zhang, X. LncRNA PVT1 accelerates LPS-induced septic acute kidney injury through targeting miR-17-5p and regulating NF-kappaB pathway. *Int Urol Nephrol* **2021**, *53*, 2409-2419, doi:https://dx.doi.org/10.1007/s11255-021-02905-8.
60. Zhang, B.; You, T.; Liu, Y.; Li, P. CIRC_0114428 INFLUENCES THE PROGRESSION OF SEPTIC ACUTE KIDNEY INJURY VIA REGULATING MIR-370-3P/TIMP2 AXIS. *Shock* **2023**, *59*, 505-513, doi:https://dx.doi.org/10.1097/SHK.0000000000002077.

61. Zhang, F.; Luo, X.; Wang, Y.; Ma, L.; Sun, D. LncRNA PMS2L2 Is Downregulated in Sepsis-Induced Acute Kidney Injury and Inhibits LPS-Induced Apoptosis of Podocytes. *Kidney Blood Press Res* **2023**, *48*, 515-521, doi:https://dx.doi.org/10.1159/000528053.
62. Zhang, H.; Che, L.; Wang, Y.; Zhou, H.; Gong, H.; Man, X.; Zhao, Q. Deregulated microRNA-22-3p in patients with sepsis-induced acute kidney injury serves as a new biomarker to predict disease occurrence and 28-day survival outcomes. *Int Urol Nephrol* **2021**, *53*, 2107-2116, doi:https://dx.doi.org/10.1007/s11255-021-02784-z.
63. Zhang, J.; Wang, C.J.; Tang, X.M.; Wei, Y.K. Urinary miR-26b as a potential biomarker for patients with sepsis-associated acute kidney injury: a Chinese population-based study. *Eur Rev Med Pharmacol Sci* **2018**, *22*, 4604-4610, doi:https://dx.doi.org/10.26355/eurrev_201807_15518.
64. Zhang, L.; Xu, Y.; Xue, S.; Wang, X.; Dai, H.; Qian, J.; Ni, Z.; Yan, Y. Implications of dynamic changes in miR-192 expression in ischemic acute kidney injury. *Int Urol Nephrol* **2017**, *49*, 541-550, doi:https://dx.doi.org/10.1007/s11255-016-1485-7.
65. Zhang, P.; Yin, J.; Xun, L.; Ding, T.; Du, S. CIRC_0002131 CONTRIBUTES TO LPS-INDUCED APOPTOSIS, INFLAMMATION, AND OXIDATIVE INJURY IN HK-2 CELLS VIA INHIBITING THE BINDING BETWEEN MIR-942-5P AND OXSR1. *Shock* **2023**, *60*, 517-524, doi:https://dx.doi.org/10.1097/SHK.0000000000002197.
66. Zhang, X.; Huang, Z.; Wang, Y.; Wang, T.; Li, J.; Xi, P. Long Non-Coding RNA RMRP Contributes to Sepsis-Induced Acute Kidney Injury. *Yonsei Med J* **2021**, *62*, 262-273, doi:https://dx.doi.org/10.3349/ymj.2021.62.3.262.
67. Zhao, H.; Chen, B.; Li, Z.; Wang, B.; Li, L. Long Noncoding RNA DANCR Suppressed Lipopolysaccharide-Induced Septic Acute Kidney Injury by Regulating miR-214 in HK-2 Cells. *Med Sci Monit* **2020**, *26*, e921822, doi:https://dx.doi.org/10.12659/MSM.921822.
68. Zheng, C.; Wu, D.; Shi, S.; Wang, L. miR-34b-5p promotes renal cell inflammation and apoptosis by inhibiting aquaporin-2 in sepsis-induced acute kidney injury. *Renal failure* **2021**, *43*, 291-301, doi:https://dx.doi.org/10.1080/0886022X.2021.1871922.
69. Zhou, F.; Liu, D.; Ye, J.; Li, B. Circ_0006944 aggravates LPS-induced HK2 cell injury via modulating miR-205-5p/UBL4A pathway. *Autoimmunity* **2023**, *56*, 2276066, doi:https://dx.doi.org/10.1080/08916934.2023.2276066.
70. Zhou, Y.; Qing, M.; Xu, M. Circ-BNIP3L knockdown alleviates LPS-induced renal tubular epithelial cell injury during sepsis-associated acute kidney injury by miR-370-3p/MYD88 axis. *J Bioenerg Biomembr* **2021**, *53*, 665-677, doi:https://dx.doi.org/10.1007/s10863-021-09925-0.
71. Zou, Y.-F.; Wen, D.; Zhao, Q.; Shen, P.-Y.; Shi, H.; Zhao, Q.; Chen, Y.-X.; Zhang, W. Urinary MicroRNA-30c-5p and MicroRNA-192-5p as potential biomarkers of ischemia-reperfusion-induced kidney injury. *Exp Biol Med (Maywood)* **2017**, *242*, 657-667, doi:https://dx.doi.org/10.1177/1535370216685005.
