# Supplementary material for: A Systematic Review and Meta-Analysis of MicroRNA as Predictive Biomarkers of Acute Kidney Injury
Source: Biomedicines. 2024 Jul 30;12(8):1695. doi: 10.3390/biomedicines12081695 (PMC11351452; doi:10.3390/biomedicines12081695)
Supplement: Supplementary file 1 [file biomedicines-12-01695-s001.zip › Table S3.pdf]

Table S3: Newcastle Ottawa Scale assessment for each included study

Case-control studies		Selection				Comparability	Exposure			Total /9
**Paper**	**Author**	1	2	3	4	1	1	2	3	
A Pilot Study Identifying a Set of microRNAs As Precise Diagnostic Biomarkers of Acute Kidney Injury	3. Aguado-Fraile et al., 2015 (n=41)	*	b	*	*	c	*	*	*	5
		AKI determined using AKIN and RIFLE criteria.	Selection criteria not stated.	Controls representative of cardiac surgery population	No AKI controls	Not matched for age/sex between groups. No comorbidity data provided.	qRT-PCR	Same method for cases and controls	All responses recorded for both groups.	Fair
Early detection of cardiac surgery-associated acute kidney injury by microRNA-21	Arvin et al., 2017	*	*	*	*	**	*	*	*	9
		AKI determined using KDIGO definition	Consecutive patient recruitment	Controls representative of cardiac surgery population	No AKI or kidney-related diseases	Study controls for age and sex. Study controls for comorbidities.	qRT-PCR	Same method for cases and controls	All responses recorded for both groups.	Very good
MicroRNA-21 and risk of severe acute kidney injury and poor outcomes after adult cardiac surgery	Du et al., 2013	*	b	*	*	**	*	*	*	8
		AKI determined using AKIN criteria.	Selection criteria not stated.	Controls representative of cardiac surgery population	No AKI or kidney-related diseases	Study controls for age and sex. Study controls for comorbidities.	qRT-PCR	Same method for cases and controls	All responses recorded for both groups.	Very good
Hsa-miR-494-3p attenuates gene HtrA3 transcription to increase inflammatory response in hypoxia/reoxygenation HK2 Cells	Gong et al., 2021	*	b	*	*	*	*	*	*	6
		AKI determined using KDIGO definition	Selection criteria not stated.	Controls representative of cardiac surgery population	No AKI controls	Study controls for age and sex. Study only controls for heart failure.	qRT-PCR	Same method for cases and controls	All responses recorded for both groups.	Good
Dysregulated microRNAs involved in contrast-induced acute kidney injury in rat and human	Gutierrez-Escolano et al., 2015	b	*	*	*	**	*	*	*	8
		Study refers to criteria but it is not considered independently validated.	Consecutive patient recruitment	Controls representative of patients undergoing coronary interventional procedures.	No AKI or kidney-related diseases	Study controls for age and sex. Study controls for comorbidities.	qRT-PCR	Same method for cases and controls	All responses recorded for both groups.	Very good

Inhibition of MiR-106b-5p mediated by exosomes mitigates acute kidney injury by modulating transmissible endoplasmic reticulum stress and M1 macrophage polarization	Li et al., 2023a	*	b	*	*	**	*	*	*	8
		AKI determined using KDIGO definition	Selection criteria not stated.	Controls representative of cardiac surgery population	No AKI or renal disease	Study controls for age and sex. Study controls for comorbidities.	qRT-PCR	Same method for cases and controls	All responses recorded for both groups.	Very good
Urinary extracellular vesicles and micro-RNA as markers of acute kidney injury after cardiac surgery	1. Miller et al., 2022<br><br>Untargeted	*	b	*	*	**	*	*	*	8
		AKI determined using KDIGO definition	Selection criteria not stated.	Controls representative of cardiac surgery population	No AKI controls	Study controls for age and sex. Study controls for comorbidities.	Sequencing	Same method for cases and controls	Same response rate between groups	Very good
	2. Miller et al., 2022<br><br>Targeted	*	b	*	*	**	*	*	*	8
		AKI determined using KDIGO definition	Selection criteria not stated.	Controls representative of cardiac surgery population	No AKI controls	Study controls for age and sex. Study controls for comorbidities.	qRT-PCR	Same method for cases and controls	Same response rate between groups	Very good
Urinary versus serum microRNAs in human oxalic acid poisoning: Contrasting signals and performance	Shihana et al., 2020	*	b	*	*	c	*	*	c	4
		AKI determined using AKIN criteria.	Selection criteria not stated.	Controls representative of nephrotoxicity population	No AKI controls	Study does not control for sex. No comorbidity data provided between groups.	qRT-PCR	Same method for cases and controls	Response rates vary with no designation	Fair
An observational cohort feasibility study to identify microvesicle and mirco-RNA biomarkers of acute kidney injury following pediatric cardiac surgery	Sullo et al., 2018	*	*	*	*	c	*	*	*	7
		AKI determined using KDIGO definition	Consecutive patient recruitment	Controls representative of cardiac surgery population	No AKI or renal disease	Study does not control for age. No comorbidity data provided between groups.	TaqMan Array	Same method for cases and controls	All responses recorded for both groups.	Good
		b	*	*	*	**	*	*	*	8

Circulating MicroRNA-188, -30a, and -30e as Early Biomarkers for Contrast-Induced Acute Kidney Injury	Sun et al., 2016	Study uses a method not considered independently validated.	Consecutive patient recruitment	Controls representative of nephrotoxicity population	No AKI or renal disease	Study controls for age and sex. Study controls for comorbidities.	qRT-PCR	Same method for cases and controls	All responses recorded for both groups.	Very good
MicroRNA-668 represses MTP18 to preserve mitochondrial dynamics in ischemic acute kidney injury	2.Wei et al., 2018 (n=62)	b	b	*	*	**	*	*	*	6
		Study uses a method not considered independently validated.	Selection criteria not stated.	Controls representative of cardiac surgery population	No AKI controls	Study controls for age and sex. Study controls for comorbidities.	qRT-PCR	Same method for cases and controls	All responses recorded for both groups.	Fair
Implications of dynamic changes in miR-192 expression in ischemic acute kidney injury	Zhang et al., 2017	*	b	*	*	**	*	*	*	8
		AKI determined using KDIGO definition	Selection criteria not stated.	Controls representative of cardiac surgery population	No AKI or renal disease	Study controls for age and sex. Study controls for comorbidities.	qRT-PCR	Same method for cases and controls	All responses recorded for both groups.	Very good
Urinary MicroRNA-30c-5p and MicroRNA-192-5p as potential biomarkers of ischemia-reperfusion-induced kidney injury	Zou et al., 2017	*	b	*	*	**	*	*	*	7
		AKI determined using KDIGO definition	Selection criteria not stated.	Controls representative of cardiac surgery population	No AKI controls	Study controls for age and sex. Study controls for comorbidities.	qRT-PCR	Same method for cases and controls	All responses recorded for both groups.	Good

Cross-sectional studies		**Selection**				**Comparability**	**Outcome**		**Total /10**
**Paper**	**Author**	1	2	3	4	1	1	2	
A Pilot Study Identifying a Set of microRNAs As Precise Diagnostic Biomarkers of Acute Kidney Injury	1.Aguado-Fraile et al., 2015 (n=9)	c	b	*	**	c	**	*	6
		Sampling strategy not stated.	Limited sample size not justified.	All responses recorded for both groups.	AKI determined using AKIN criteria	Study takes into account age and sex but does not match.<br>No comorbidity data provided.	qRT-PCR	Appropriate and clearly described statistics.	Fair
	2.Aguado-Fraile et al., 2015 (n=55)	c	b	*	**	c	**	*	6
		Sampling strategy not stated.	Limited sample size not justified.	All responses recorded for both groups.	AKI determined using AKIN criteria	Study takes into account age and sex but does not match.<br>No comorbidity data provided.	qRT-PCR	Appropriate and clearly described statistics.	Fair
MicroRNA expression profiling in acute kidney injury	Aomatsu et al., 2022	c	b	*	**	*	**	*	7
		Sampling strategy not stated.	Limited sample size not justified.	All responses recorded for both groups.	AKI determined using KDIGO definition	Study controls for age and sex.<br>No comorbidity data provided.	qRT-PCR	Appropriate and clearly described statistics.	Good
Urinary miR-16 transactivated by C/EBPβ reduces kidney function after ischemia/reperfusion-induced injury	Chen et al., 2016	c	b	c	**	*	**	b	5
		Sampling strategy not stated.	Limited sample size not justified.	Less data completeness in control group.	AKI determined using RIFLE-AKIN criteria	Study controls for age and sex.<br>Not matched for comorbidities between groups.	qRT-PCR	Measure of SEM not presented on figures.	Fair
Circ_0001806 relieves LPS-induced HK2 cell injury by regulating the expression of miR-942-5p and TXNIP	Chen et al., 2023	c	b	*	c	*	**	*	5
		Sampling strategy not stated.	Limited sample size not justified.	All responses recorded for both groups.	No description of validated AKI diagnosis criteria.	Study controls for age and sex.<br>No comorbidity data provided between groups.	qRT-PCR	Appropriate and clearly described statistics.	Fair
		c	b	*	**	**	**	*	8

A circulating miRNA signature for early diagnosis of acute kidney injury following acute myocardial infarction	Fan et al., 2019	Sampling strategy not stated.	Limited sample size not justified.	All responses recorded for both groups.	AKI determined using KDIGO definition	Study controls for age and sex.<br>Study controls for comorbidities.	qRT-PCR	Appropriate and clearly described statistics.	Good
NEAT1 aggravates sepsis-induced acute kidney injury by sponging miR-22-3p	Feng et al., 2020	c	b	*	c	c	**	*	4
		Sampling strategy not stated.	Limited sample size not justified.	All responses recorded for both groups.	No description of validated AKI diagnosis criteria.	Study does not provide patient demographic data.	qRT-PCR	Appropriate and clearly described statistics.	Poor
ircSTRN3 aggravates sepsis-induced acute kidney injury by regulating miR-578/ toll like receptor 4 axis	Gao et al., 2022	c	b	*	c	c	**	*	4
		Sampling strategy not stated.	Limited sample size not justified.	All responses recorded for both groups.	No description of validated AKI diagnosis criteria.	Study does not provide patient demographic data.	qRT-PCR	Appropriate and clearly described statistics.	Poor
Differentially expressed miRNAs in sepsis- induced acute kidney injury target oxidative stress and mitochondrial dysfunction pathways	1.Ge et al., 2017<br>Untargeted (n = 55)	*	b	*	**	c	**	*	7
		Consecutive patient recruitment	Limited sample size not justified.	All responses recorded for both groups.	AKI determined using KDIGO definition	Study does not provide patient demographic data.	Microarray	Appropriate and clearly described statistics.	Good
	2.Ge et al., 2017<br><br>Targeted (n = 9)	*	b	*	**	*	**	*	8
		Consecutive patient recruitment	Limited sample size not justified.	All responses recorded for both groups.	AKI determined using KDIGO definition	Study controls for age and sex.<br>No comorbidity data provided between groups.	qRT-PCR	Appropriate and clearly described statistics.	Good
Downregulation of circ-ZNF644 alleviates LPS-induced HK2 cell injury via miR-335-5p/HIPK1 axis	Gong et al., 2022	c	b	*	**	*	**	*	7
		Sampling strategy not stated.	Limited sample size not justified.	All responses recorded for both groups.	AKI determined using AKIN criteria	Study controls for age and sex.<br>No comorbidity data provided between groups.	qRT-PCR	Appropriate and clearly described statistics.	Good
		c	b	*	**	*	**	*	7

Mechanism of circHIPK3-miRNA-124-3p/miRNA-148b-3p-Mediated Inflammatory Responses and Cell Senescence in Candida albicans -Induced Septic Acute Kidney Injury	Han et al., 2022	Sampling strategy not stated.	Limited sample size not justified.	All responses recorded for both groups.	AKI determined using KDIGO definition	Study controls for age and sex.<br>No comorbidity data provided between groups.	qRT-PCR	Appropriate and clearly described statistics.	Good
LncRNA NEAT1 promotes hypoxia-induced renal tubular epithelial apoptosis through downregulating miR-27a-3p	Jiang et al., 2019	c	b	*	c	c	**	*	4
		Sampling strategy not stated.	Limited sample size not justified.	All responses recorded for both groups.	No description of validated AKI diagnosis criteria.	Study does not provide patient demographic data.	qRT-PCR	Appropriate and clearly described statistics.	Poor
CIRC_0001818 TARGETS MIR-136-5P TO INCREASE LIPOPOLYSACCHARIDE-INDUCED HK2 CELL INJURIES BY ACTIVATING TXNIP/NLRP3 INFLAMMASOME PATHWAY	Kuang et al., 2023	c	b	*	b	*	**	*	5
		Sampling strategy not stated.	Limited sample size not justified.	All responses recorded for both groups.	Study uses a method not considered independently validated.	Study controls for age and sex.<br>No comorbidity data provided between groups.	qRT-PCR	Appropriate and clearly described statistics.	Fair
MicroRNA-494 Reduces ATF3 Expression and Promotes AKI	Lan et al., 2012	c	b	*	**	c	**	b	5
		Sampling strategy not stated.	Limited sample size not justified.	All responses recorded for both groups.	AKI determined using AKIN criteria	Study does not provide demographic data for healthy controls.	qRT-PCR	Presentation of mean SEM values on figures are unclear.	Fair
Knockdown of circ-FANCA alleviates LPS-induced HK2 cell injury via targeting miR-93-5p/OXSR1 axis in septic acute kidney injury	Li et al., 2021	c	b	*	c	*	**	*	5
		Sampling strategy not stated.	Limited sample size not justified.	All responses recorded for both groups.	No description of validated AKI diagnosis criteria.	Study controls for age and sex.<br>No comorbidity data provided between groups.	qRT-PCR	Appropriate and clearly described statistics.	Fair
Circ_0040994 depletion alleviates lipopolysaccharide-induced HK2 cell injury through miR-17-5p/TRPM7 axis	Li et al., 2023	c	b	*	c	c	**	b	3
		Sampling strategy not stated.	Limited sample size not justified.	All responses recorded for both groups.	No description of validated AKI diagnosis criteria.	Study does not provide demographic data for healthy controls.	qRT-PCR	Presentation of data not described	Poor
	Li et al., 2023b	c	b	*	c	*	**	*	5

Inhibition of sepsis-induced acute kidney injury via the circITCH-miR-579-3p-ZEB2 axis		Sampling strategy not stated.	Limited sample size not justified.	All responses recorded for both groups.	No description of validated AKI diagnosis criteria.	Study controls for age and sex.<br>No comorbidity data provided between groups.	qRT-PCR	Appropriate and clearly described statistics.	Fair
CircNRIP1 KNOCKDOWN ALLEVIATES LIPOPOLYSACCHARIDE-INDUCED HUMAN KIDNEY 2 CELL APOPTOSIS AND INFLAMMATION THROUGH miR-339-5p/OXSR1 PATHWAY	Li et al., 2023c	c	b	*	c	*	**	*	5
		Sampling strategy not stated.	Limited sample size not justified.	All responses recorded for both groups.	No description of validated AKI diagnosis criteria.	Study controls for age and sex.<br>No comorbidity data provided between groups.	qRT-PCR	Appropriate and clearly described statistics.	Fair
Expression patterns and prognostic value of miR-210, miR-494, and miR-205 in middle-aged and old patients with sepsis-induced acute kidney injury.	Lin et al., 2019	c	*	*	**	**	**	*	9
		Sampling strategy not stated.	Satisfactory sample size (n = 220)	All responses recorded for both groups.	AKI determined using AKIN criteria	Study controls for age and sex.<br>Study controls for comorbidities.	TaqMan low density array	Appropriate and clearly described statistics.	Very good
Discovery and validation of miR-452 as an effective biomarker for acute kidney injury in sepsis	Liu et al., 2020	c	b	*	**	*	**	*	7
		Sampling strategy not stated.	Limited sample size not justified.	All responses recorded for both groups.	AKI determined using KDIGO definition	Study controls for age and sex.<br>No comorbidity data provided between groups.	qRT-PCR	Appropriate and clearly described statistics.	Good
The negative feedback loop of NF-kappaB/miR-376b/NFKBIZ in septic acute kidney injury	Liu et al., 2020a	c	b	*	**	*	**	*	7
		Sampling strategy not stated.	Limited sample size not justified.	All responses recorded for both groups.	AKI determined using KDIGO definition	Study controls for age and sex.<br>No comorbidity data provided between groups.	qRT-PCR	Appropriate and clearly described statistics.	Good
Downregulation of miR-574-5p inhibits HK-2 cell viability and predicts the onset of acute kidney injury in sepsis patients	Liu et al., 2021	c	*	*	**	*	**	*	8
		Sampling strategy not stated.	Satisfactory sample size (n = 136)	All responses recorded for both groups.	AKI determined using KDIGO definition.	Study controls for age and sex.<br>Comorbidity data provided but healthy controls have sepsis.	qRT-PCR	Appropriate and clearly described statistics.	Good

Exosomal microRNA-342-5p secreted from adipose-derived mesenchymal stem cells mitigates acute kidney injury in sepsis mice by inhibiting TLR13	Liu et al., 2023	*	b	*	**	*	**	*	8
		Consecutive patient recruitment	Limited sample size not justified.	All responses recorded for both groups.	AKI determined using KDIGO definition	Study controls for age and sex. No comorbidity data provided between groups.	qRT-PCR	Appropriate and clearly described statistics.	Good
Circulating miR-210 Predicts Survival in Critically Ill Patients with Acute Kidney Injury	1.Lorenzen et al., 2011 Untargeted (n=10)	c	b	*	**	c	**	b	5
		Sampling strategy not stated.	Limited sample size not justified.	All responses recorded for both groups.	AKI determined using RIFLE criteria	Study does not provide patient demographic data.	GeneChip miRNA arrays	Statistical test for fold-change not described.	Fair
	2.Lorenzen et al., 2011 Targeted (n=107)	c	*	*	**	c	**	*	7
		Sampling strategy not stated.	Satisfactory sample size (n = 107)	All responses recorded for both groups.	AKI determined using RIFLE criteria	Study does not provide demographic data for healthy controls.	qRT-PCR	Appropriate and clearly described statistics.	Good
SIKIAT1/miR-96/FOXA1 axis regulates sepsis-induced kidney injury through induction of apoptosis	Lu et al., 2020	c	b	*	c	c	**	*	4
		Sampling strategy not stated.	Limited sample size not justified.	All responses recorded for both groups.	No description of validated AKI diagnosis criteria.	Study does not provide patient demographic data.	qRT-PCR	Appropriate and clearly described statistics.	Poor
Circular RNA HIPK3 aggravates sepsis-induced acute kidney injury via modulating the microRNA-338/forkhead box A1 axis	Lu et al., 2022	c	b	*	c	c	**	*	4
		Sampling strategy not stated.	Limited sample size not justified.	All responses recorded for both groups.	No description of validated AKI diagnosis criteria.	Study does not provide patient demographic data.	qRT-PCR	Appropriate and clearly described statistics.	Poor
A novel role of the miR-152-3p/ERRFI1/STAT3 pathway modulates the apoptosis and inflammatory response after acute kidney injury	Ma et al., 2020	c	b	*	c	*	**	*	5
		Sampling strategy not stated.	Limited sample size not justified.	All responses recorded for both groups.	No description of validated AKI diagnosis criteria.	Study controls for age and sex. No comorbidity data provided between groups.	qRT-PCR	Appropriate and clearly described statistics.	Fair
	Ma et al., 2022	c	b	*	**	**	**	b	7

The Potential of miR-370-3p and miR-495-3p Serving as Biomarkers for Sepsis-Associated Acute Kidney Injury		Sampling strategy not stated.	Limited sample size not justified.	All responses recorded for both groups.	AKI determined using KDIGO-RIFLE criteria	Study controls for age and sex.<br>Study controls for comorbidities.	qRT-PCR	Presentation of data not described	Good
MEG3 aggravates hypoxia/reoxygenation induced apoptosis of renal tubular epithelial cells via the miR-129-5p/HMGB1 axis	Mao et al., 2021	c	b	*	c	c	**	*	4
		Sampling strategy not stated.	Limited sample size not justified.	All responses recorded for both groups.	No description of validated AKI diagnosis criteria.	Study does not provide patient demographic data.	qRT-PCR	Appropriate and clearly described statistics.	Poor
miR-141 mediates recovery from acute kidney injury	Newbury et al., 2021	c	b	*	**	c	**	*	6
		Sampling strategy not stated.	Limited sample size not justified.	All responses recorded for both groups.	AKI determined using KDIGO definition	Study does not provide demographic data between AKI and control.	qRT-PCR	Appropriate and clearly described statistics.	Fair
Detection of Drug-Induced Acute Kidney Injury in Humans Using Urinary KIM-1, miR-21, -200c, and -423	Pavkovic et al., 2016	c	*	*	b	*	**	*	6
		Sampling strategy not stated.	Satisfactory sample size (n = 108)	All responses recorded for both groups.	Study refers to criteria but it is not considered independently validated.	Study controls for age and sex.<br>No comorbidity data provided between groups.	qRT-PCR	Appropriate and clearly described statistics.	Fair
Human miRNome Profiling Identifies MicroRNAs Differentially Present in the Urine after Kidney Injury	Ramachandran et al., 2013	c	*	*	**	*	**	*	8
		Sampling strategy not stated.	Satisfactory sample size (n = 145)	All responses recorded for both groups.	AKI determined using KDIGO definition	Study controls for age and sex.<br>No comorbidity data provided between groups.	qRT-PCR	Appropriate and clearly described statistics.	Good
Expression, circulation and excretion profile of miRNA-21, -155 and -18a following acute kidney injury	Saikumar et al., 2012	c	b	*	c	c	**	*	4
		Sampling strategy not stated.	Limited sample size not justified.	All responses recorded for both groups.	No description of validated AKI diagnosis criteria.	Study does not control for age and sex.<br>No comorbidity data provided between groups.	qRT-PCR	Appropriate and clearly described statistics.	Poor
	Shi et al., 2021	c	b	*	**	c	**	b	5

MiR-150-5p protects against septic acute kidney injury via repressing the MEKK3/JNK pathway		Sampling strategy not stated.	Limited sample size not justified.	All responses recorded for both groups.	AKI determined using KDIGO definition	Study does not control for sex.<br>No comorbidity data provided between groups.	qRT-PCR	Measure of data variability not presented on figures.	Fair
Correlation Between Single Nucleotide Polymorphisms at the 3'-UTR of the NFKB1 Gene and Acute Kidney Injury in Sepsis	Sun et al., 2020	c	*	*	b	*	**	*	6
		Sampling strategy not stated.	Satisfactory sample size (n = 470)	All responses recorded for both groups.	Study refers to criteria but it is not considered independently validated.	Study controls for age and sex.<br>No comorbidity data provided between groups.	qRT-PCR	Appropriate and clearly described statistics.	Fair
Circ_0091702 serves as a sponge of miR-545-3p to attenuate sepsis-related acute kidney injury by upregulating THBS2	Tan et al., 2021	c	b	*	c	*	**	*	5
		Sampling strategy not stated.	Limited sample size not justified.	All responses recorded for both groups.	No description of validated AKI diagnosis criteria.	Study controls for age and sex.<br>No comorbidity data provided between groups.	qRT-PCR	Appropriate and clearly described statistics.	Fair
Downregulation of XIST ameliorates acute kidney injury by sponging miR-142-5p and targeting PDCD4	Tang et al., 2020	c	*	*	c	c	**	*	5
		Sampling strategy not stated.	Satisfactory sample size (n = 200)	All responses recorded for both groups.	No description of validated AKI diagnosis criteria.	Study does not provide patient demographic data.	qRT-PCR	Appropriate and clearly described statistics.	Fair
MiR-107 induces TNF-alpha secretion in endothelial cells causing tubular cell injury in patients with septic acute kidney injury	Wang et al., 2017	c	b	*	**	*	**	*	7
		Sampling strategy not stated.	Limited sample size not justified.	All responses recorded for both groups.	AKI determined using KDIGO definition	Study controls for age and sex.<br>No comorbidity data provided between groups.	qRT-PCR	Appropriate and clearly described statistics.	Good
Down-regulation of lncRNA SNHG5 relieves sepsis-induced acute kidney injury by regulating the miR-374a-3p/TLR4/NF-κB pathway	Wang et al., 2021	c	*	*	c	*	**	*	6
		Sampling strategy not stated.	Satisfactory sample size (n = 105)	All responses recorded for both groups.	No description of validated AKI diagnosis criteria.	Study controls for age and sex.<br>No comorbidity data provided between groups.	qRT-PCR	Appropriate and clearly described statistics.	Fair
		c	b	*	c	c	**	*	4

Silencing circ_0074371 inhibits the progression of sepsis-induced acute kidney injury by regulating miR-330-5p/ELK1 axis	Wang et al., 2022	Sampling strategy not stated.	Limited sample size not justified.	All responses recorded for both groups.	No description of validated AKI diagnosis criteria.	Study does not provide patient demographic data.	qRT-PCR	Appropriate and clearly described statistics.	Poor
microRNA-338-3p suppresses lipopolysaccharide-induced inflammatory response in HK-2 cells	Wang et al., 2022a	c	b	c	c	c	**	b	2
		Sampling strategy not stated.	Limited sample size not justified.	Study does not state population size	No description of validated AKI diagnosis criteria.	Study does not provide patient demographic data.	qRT-PCR	Presentation of data not described	Poor
CircVMA21 ameliorates lipopolysaccharide (LPS)-induced HK-2 cell injury depending on the regulation of miR-7-5p/PPARA.	Wang et al., 2022b	c	b	*	c	c	**	*	4
		Sampling strategy not stated.	Limited sample size not justified.	All responses recorded for both groups.	No description of validated AKI diagnosis criteria.	Study does not provide patient demographic data.	qRT-PCR	Appropriate and clearly described statistics.	Poor
MicroRNA-668 represses MTP18 to preserve mitochondrial dynamics in ischemic acute kidney injury	1.Wei et al., 2018 (n=16)	c	b	*	c	**	**	*	6
		Sampling strategy not stated.	Limited sample size not justified.	All responses recorded for both groups.	No description of validated AKI diagnosis criteria.	Study controls for age and sex. Study controls for comorbidities.	qRT-PCR	Appropriate and clearly described statistics.	Fair
The miR-15a-5p-XIST-CUL3 regulatory axis is important for sepsis-induced acute kidney injury	Xu et al., 2019	c	b	*	c	*	**	*	5
		Sampling strategy not stated.	Limited sample size not justified.	All responses recorded for both groups.	No description of validated AKI diagnosis criteria.	Study controls for age and sex. No comorbidity data provided between groups.	qRT-PCR	Appropriate and clearly described statistics.	Fair
miR-195-5p alleviates acute kidney injury through repression of inflammation and oxidative stress by targeting vascular endothelial growth factor A	Xu et al., 2020	c	*	*	c	c	**	*	5
		Sampling strategy not stated.	Satisfactory sample size (n = 160)	All responses recorded for both groups.	No description of validated AKI diagnosis criteria.	Study does not provide patient demographic data.	qRT-PCR	Appropriate and clearly described statistics.	Fair
	Xu et al., 2022	c	b	*	c	*	**	*	5

Circ_0114427 promotes LPS-induced septic acute kidney injury by modulating miR-495-3p/TRAF6 through the NF-κB pathway		Sampling strategy not stated.	Limited sample size not justified.	All responses recorded for both groups.	No description of validated AKI diagnosis criteria.	Study controls for age and sex. No comorbidity data provided between groups.	qRT-PCR	Appropriate and clearly described statistics.	Fair
THE VALUE OF COMBINING MIR-10A-5P LEVELS AND PLR TO EVALUATE THE PROGNOSIS OF SEPSIS PATIENTS WITH ACUTE KIDNEY INJURY	Xun et al., 2022	c	*	*	c	*	b	b	3
		Sampling strategy not stated.	Satisfactory sample size (n = 142)	All responses recorded for both groups.	No description of validated AKI diagnosis criteria.	Study controls for age and sex. No comorbidity data provided between groups.	No description of non-standard 'PT-PCR' method	Tables missing units.	Poor
Long non-coding RNA SNHG14 aggravates LPS-induced acute kidney injury through regulating miR-495-3p/HIPK1	Yang et al., 2021	c	b	*	c	c	**	*	4
		Sampling strategy not stated.	Limited sample size not justified.	All responses recorded for both groups.	No description of validated AKI diagnosis criteria.	Study does not provide patient demographic data.	qRT-PCR	Appropriate and clearly described statistics.	Fair
miR-23a-3p inhibits sepsis-induced kidney epithelial cell injury by suppressing Wnt/beta-catenin signaling by targeting wnt5a	Ye et al., 2022	c	b	*	**	*	**	*	7
		Sampling strategy not stated.	Limited sample size not justified.	All responses recorded for both groups.	AKI determined using AKIN criteria	Study controls for age and sex. No comorbidity data provided between groups.	qRT-PCR	Appropriate and clearly described statistics.	Good
CIRC_0008882 STIMULATES PDE7A TO SUPPRESS SEPTIC ACUTE KIDNEY INJURY PROGRESSION BY SPONGING MIR-155-5P	You et al., 2023	c	b	*	c	*	**	*	5
		Sampling strategy not stated.	Limited sample size not justified.	All responses recorded for both groups.	No description of validated AKI diagnosis criteria.	Study controls for age and sex. No comorbidity data provided.	qRT-PCR	Appropriate and clearly described statistics.	Fair
LncRNA PVT1 accelerates LPS-induced septic acute kidney injury through targeting miR-17-5p and regulating NF-kappaB pathway	Yuan et al., 2021	c	b	*	**	*	**	*	7
		Sampling strategy not stated.	Limited sample size not justified.	All responses recorded for both groups.	AKI determined using KDIGO definition	Study controls for age and sex. No comorbidity data provided.	qRT-PCR	Appropriate and clearly described statistics.	Good

Urinary miR-26b as a potential biomarker for patients with sepsis-associated acute kidney injury: a Chinese population-based study	Zhang et al., 2018	*	*	*	**	c	**	*	8
		Consecutive patient recruitment	Satisfactory sample size (n = 155)	All responses recorded for both groups.	AKI determined using KDIGO definition	Study does not provide patient demographic data.	qRT-PCR	Appropriate and clearly described statistics.	Good
Long Non-Coding RNA RMRP Contributes to Sepsis-Induced Acute Kidney Injury	Zhang et al., 2021	c	b	*	c	c	**	*	4
		Sampling strategy not stated.	Limited sample size not justified.	All responses recorded for both groups.	No description of validated AKI diagnosis criteria.	Study does not provide patient demographic data.	qRT-PCR	Appropriate and clearly described statistics.	Poor
Deregulated microRNA-22-3p in patients with sepsis-induced acute kidney injury serves as a new biomarker to predict disease occurrence and 28-day survival outcomes	Zhang et al., 2021a	c	*	*	**	**	**	*	9
		Sampling strategy not stated.	Satisfactory sample size (n = 158)	All responses recorded for both groups.	AKI determined using KDIGO definition	Study controls for age and sex. Study controls for comorbidities.	qRT-PCR	Appropriate and clearly described statistics.	Very good
CIRC_0002131 CONTRIBUTES TO LPS-INDUCED APOPTOSIS, INFLAMMATION, AND OXIDATIVE INJURY IN HK-2 CELLS VIA INHIBITING THE BINDING BETWEEN MIR-942-5P AND OXSR5	Zhang et al., 2023	c	b	*	c	*	**	*	5
		Sampling strategy not stated.	Limited sample size not justified.	All responses recorded for both groups.	No description of validated AKI diagnosis criteria.	Study controls for age and sex. No comorbidity data provided.	qRT-PCR	Appropriate and clearly described statistics.	Fair
LncRNA PMS2L2 Is Downregulated in Sepsis-Induced Acute Kidney Injury and Inhibits LPS-Induced Apoptosis of podocytes	Zhang et al., 2023a	c	*	*	b	*	**	b	5
		Sampling strategy not stated.	Satisfactory sample size (n = 100)	All responses recorded for both groups.	Study uses a method not considered independently validated.	Study controls for age and sex. No comorbidity data provided.	qRT-PCR	Presentation of data not described	Fair
CIRC_0114428 INFLUENCES THE PROGRESSION OF SEPTIC ACUTE KIDNEY INJURY VIA REGULATING MIR-370-3P/TIMP2 AXIS	Zhang et al., 2023b	c	b	*	c	*	**	*	5
		Sampling strategy not stated.	Limited sample size not justified.	All responses recorded for both groups.	No description of validated AKI diagnosis criteria.	Study controls for age and sex. No comorbidity data provided.	qRT-PCR	Appropriate and clearly described statistics.	Fair

Long Noncoding RNA DANCR Suppressed Lipopolysaccharide-Induced Septic Acute Kidney Injury by Regulating miR-214 in HK-2 Cells	Zhao et al., 2020	c	b	*	**	*	**	*	7
		Sampling strategy not stated.	Limited sample size not justified.	All responses recorded for both groups.	AKI determined using AKIN criteria	Study controls for age and sex. No comorbidity data provided.	qRT-PCR	Appropriate and clearly described statistics.	Good
miR-34b-5p promotes renal cell inflammation and apoptosis by inhibiting aquaporin-2 in sepsis-induced acute kidney injury.	Zheng et al., 2021	c	b	*	**	*	**	*	7
		Sampling strategy not stated.	Limited sample size not justified.	All responses recorded for both groups.	AKI determined using KDIGO definition	Study controls for age and sex. No comorbidity data provided.	qRT-PCR	Appropriate and clearly described statistics.	Good
Circ-BNIP3L knockdown alleviates LPS-induced renal tubular epithelial cell injury during sepsis-associated acute kidney injury by miR-370-3p/MYD88 axis	Zhou et al., 2021	c	b	*	**	*	**	*	7
		Sampling strategy not stated.	Limited sample size not justified.	All responses recorded for both groups.	AKI determined using AKIN criteria	Study controls for age and sex. No comorbidity data provided.	qRT-PCR	Appropriate and clearly described statistics.	Good
Circ_0006944 aggravates LPS-induced HK2 cell injury via modulating miR-205-5p/UBL4A pathway	Zhou et al., 2023	c	b	*	c	c	**	*	4
		Sampling strategy not stated.	Limited sample size not justified.	All responses recorded for both groups.	No description of validated AKI diagnosis criteria.	Study does not provide patient demographic data.	qRT-PCR	Appropriate and clearly described statistics.	Poor

1. Aguado-Fraile, E.; Ramos, E.; Conde, E.; Rodríguez, M.; Martín-Gómez, L.; Lietor, A.; Candela, Á.; Ponte, B.; Liaño, F.; García-Bermejo, M.L. A Pilot Study Identifying a Set of microRNAs As Precise Diagnostic Biomarkers of Acute Kidney Injury. *PLoS ONE* **2015**, *10*, e0127175, doi:10.1371/journal.pone.0127175.
2. Aomatsu, A.; Kaneko, S.; Yanai, K.; Ishii, H.; Ito, K.; Hirai, K.; Ookawara, S.; Kobayashi, Y.; Sanui, M.; Morishita, Y. MicroRNA expression profiling in acute kidney injury. *Transl Res* **2022**, *244*, 1-31, doi:https://dx.doi.org/10.1016/j.trsl.2021.11.010.
3. Arvin, P.; Samimagham, H.R.; Montazerghaem, H.; Khayatian, M.; Mahboobi, H.; Ghadiri Soufi, F. Early detection of cardiac surgery-associated acute kidney injury by microRNA-21. *Bratisl Lek Listy* **2017**, *118*, 626-631, doi:https://dx.doi.org/10.4149/BLL_2017_120.
4. Chen, H.H.; Lan, Y.F.; Li, H.F.; Cheng, C.F.; Lai, P.F.; Li, W.H.; Lin, H. Urinary miR-16 transactivated by C/EBPβ reduces kidney function after ischemia/reperfusion-induced injury. *Scientific reports* **2016**, *6*, 27945, doi:10.1038/srep27945.
5. Chen, M.; Zhang, L. Circ_0001806 relieves LPS-induced HK2 cell injury by regulating the expression of miR-942-5p and TXNIP. *J Bioenerg Biomembr* **2023**, *55*, 301-312, doi:https://dx.doi.org/10.1007/s10863-023-09978-3.
6. Du, J.; Cao, X.; Zou, L.; Chen, Y.; Guo, J.; Chen, Z.; Hu, S.; Zheng, Z. MicroRNA-21 and risk of severe acute kidney injury and poor outcomes after adult cardiac surgery. *PLoS ONE* **2013**, *8*, e63390, doi:https://dx.doi.org/10.1371/journal.pone.0063390.
7. Fan, P.C.; Chen, C.C.; Peng, C.C.; Chang, C.H.; Yang, C.H.; Yang, C.; Chu, L.J.; Chen, Y.C.; Yang, C.W.; Chang, Y.S.; Chu, P.H. A circulating miRNA signature for early diagnosis of acute kidney injury following acute myocardial infarction. *J Transl Med* **2019**, *17*, 139, doi:10.1186/s12967-019-1890-7.
8. Feng, Y.; Liu, J.; Wu, R.; Yang, P.; Ye, Z.; Song, F. NEAT1 aggravates sepsis-induced acute kidney injury by sponging miR-22-3p. **2020**, *15*, 333-342, doi:doi:10.1515/med-2020-0401.
9. Gao, Q.; Zheng, Y.; Wang, H.; Hou, L.; Hu, X. circSTRN3 aggravates sepsis-induced acute kidney injury by regulating miR-578/ toll like receptor 4 axis. *Bioengineered* **2022**, *13*, 11388-11401, doi:10.1080/21655979.2022.2061293.
10. Ge, Q.M.; Huang, C.M.; Zhu, X.Y.; Bian, F.; Pan, S.M. Differentially expressed miRNAs in sepsis-induced acute kidney injury target oxidative stress and mitochondrial dysfunction pathways. *PLoS ONE* **2017**, *12*, e0173292, doi:10.1371/journal.pone.0173292.
11. Gong, J.; Zhao, S.; Luo, S.; Yin, S.; Li, X.; Feng, Y. Downregulation of circ-ZNF644 alleviates LPS-induced HK2 cell injury via miR-335-5p/HIPK1 axis. *Environ Toxicol* **2022**, *37*, 2855-2864, doi:https://dx.doi.org/10.1002/tox.23642.
12. Gong, Q.; Shen, Z.-M.; Sheng, Z.; Jiang, S.; Ge, S.-L. Hsa-miR-494-3p attenuates gene HtrA3 transcription to increase inflammatory response in hypoxia/reoxygenation HK2 Cells. *Scientific reports* **2021**, *11*, 1665, doi:https://dx.doi.org/10.1038/s41598-021-81113-x.
13. Gutiérrez-Escolano, A.; Santacruz-Vázquez, E.; Gómez-Pérez, F. Dysregulated microRNAs involved in contrast-induced acute kidney injury in rat and human. *Renal Failure* **2015**, *37*, 1498-1506, doi:10.3109/0886022X.2015.1077322.
14. Han, J.; Li, W.; Zhang, J.; Guan, Y.; Huang, Y.; Li, X. Mechanism of circHIPK3-miRNA-124-3p/miRNA-148b-3p-Mediated Inflammatory Responses and Cell Senescence in Candida albicans -Induced Septic Acute Kidney Injury. *Gerontology* **2022**, *68*, 1145-1165, doi:10.1159/000523910.
15. Jiang, X.; Li, D.; Shen, W.; Shen, X.; Liu, Y. LncRNA NEAT1 promotes hypoxia-induced renal tubular epithelial apoptosis through downregulating miR-27a-3p. *J Cell Biochem* **2019**, *120*, 16273-16282, doi:https://dx.doi.org/10.1002/jcb.28909.
16. Kuang, F.; Wang, B.; You, T.; Liu, Y.; Li, P.; Wang, J.; Peng, L. CIRC_0001818 TARGETS MIR-136-5P TO INCREASE LIPOPOLYSACCHARIDE-INDUCED HK2 CELL INJURIES BY ACTIVATING TXNIP/NLRP3 INFLAMMASOME PATHWAY. *Shock* **2023**, *60*, 110-120, doi:https://dx.doi.org/10.1097/SHK.0000000000002140.
17. Lan, Y.-F.; Chen, H.-H.; Lai, P.-F.; Cheng, C.-F.; Huang, Y.-T.; Lee, Y.-C.; Chen, T.-W.; Lin, H. MicroRNA-494 reduces ATF3 expression and promotes AKI. *J Am Soc Nephrol* **2012**, *23*, 2012-2023, doi:https://dx.doi.org/10.1681/ASN.2012050438.
18. Li, H.; Zhang, X.; Wang, P.; Zhou, X.; Liang, H.; Li, C. Knockdown of circ-FANCA alleviates LPS-induced HK2 cell injury via targeting miR-93-5p/OXSR1 axis in septic acute kidney injury. *Diabetology and Metabolic Syndrome* **2021**, *13*, doi:10.1186/s13098-021-00625-8.
19. Li, P.; Liu, Y.; You, T. CircNRIP1 KNOCKDOWN ALLEVIATES LIPOPOLYSACCHARIDE-INDUCED HUMAN KIDNEY 2 CELL APOPTOSIS AND INFLAMMATION THROUGH miR-339-5p/OXSR1 PATHWAY. *Shock* **2023**, *59*, 426-433, doi:https://dx.doi.org/10.1097/SHK.0000000000002057.

20. Li, Q.; Wang, T.; Wang, X.; Ge, X.-Y.; Yang, T.; Bai, G.; Wang, W. Inhibition of sepsis-induced acute kidney injury via the circITCH-miR-579-3p-ZEB2 axis. *Environ Toxicol* **2023**, *38*, 1217-1225, doi:https://dx.doi.org/10.1002/tox.23682.
21. Li, X.; Zhong, Y.; Yue, R.; Xie, J.; Zhang, Y.; Lin, Y.; Li, H.; Xu, Y.; Zheng, D. Inhibition of MiR-106b-5p mediated by exosomes mitigates acute kidney injury by modulating transmissible endoplasmic reticulum stress and M1 macrophage polarization. *Journal of Cellular and Molecular Medicine* **2023**, *27*, 2876-2889, doi:10.1111/jcmm.17848.
22. Li, Y.; Chai, Y. Circ_0040994 depletion alleviates lipopolysaccharide-induced HK2 cell injury through miR-17-5p/TRPM7 axis. *Environ Toxicol* **2023**, *38*, 2585-2594, doi:https://dx.doi.org/10.1002/tox.23894.
23. Lin, Y.; Ding, Y.; Song, S.; Li, M.; Wang, T.; Guo, F. Expression patterns and prognostic value of miR-210, miR-494, and miR-205 in middle-aged and old patients with sepsis-induced acute kidney injury. *Bosn. j. basic med. sci.* **2019**, *19*, 249-256, doi:https://dx.doi.org/10.17305/bjbms.2019.4131.
24. Liu, S.; Zhao, L.; Zhang, L.; Qiao, L.; Gao, S. Downregulation of miR-574-5p inhibits HK-2 cell viability and predicts the onset of acute kidney injury in sepsis patients. *Renal failure* **2021**, *43*, 942-948, doi:https://dx.doi.org/10.1080/0886022X.2021.1939051.
25. Liu, W.; Hu, C.; Zhang, B.; Li, M.; Deng, F.; Zhao, S. Exosomal microRNA-342-5p secreted from adipose-derived mesenchymal stem cells mitigates acute kidney injury in sepsis mice by inhibiting TLR9. *Biol. proced. online* **2023**, *25*, 10, doi:https://dx.doi.org/10.1186/s12575-023-00198-y.
26. Liu, Z.; Tang, C.; He, L.; Yang, D.; Cai, J.; Zhu, J.; Shu, S.; Liu, Y.; Yin, L.; Chen, G.; et al. The negative feedback loop of NF-kappaB/miR-376b/NFKBIZ in septic acute kidney injury. *JCI insight* **2020**, *5*, doi:https://dx.doi.org/10.1172/jci.insight.142272.
27. Liu, Z.; Yang, D.; Gao, J.; Xiang, X.; Hu, X.; Li, S.; Wu, W.; Cai, J.; Tang, C.; Zhang, D.; Dong, Z. Discovery and validation of miR-452 as an effective biomarker for acute kidney injury in sepsis. *Theranostics* **2020**, *10*, 11963-11975, doi:https://dx.doi.org/10.7150/thno.50093.
28. Lorenzen, J.M.; Kielstein, J.T.; Hafer, C.; Gupta, S.K.; Kümpers, P.; Faulhaber-Walter, R.; Haller, H.; Fliser, D.; Thum, T. Circulating miR-210 Predicts Survival in Critically Ill Patients with Acute Kidney Injury. *Clinical Journal of the American Society of Nephrology* **2011**, *6*.
29. Lu, H.; Chen, Y.; Wang, X.; Yang, Y.; Ding, M.; Qiu, F. Circular RNA HIPK3 aggravates sepsis-induced acute kidney injury via modulating the microRNA-338/forkhead box A1 axis. *Bioengineered* **2022**, *13*, 4798-4809, doi:https://dx.doi.org/10.1080/21655979.2022.2032974.
30. Lu, S.; Wu, H.; Xu, J.; He, Z.; Li, H.; Ning, C. SIKIAT1/miR-96/FOXA1 axis regulates sepsis-induced kidney injury through induction of apoptosis. *Inflamm Res* **2020**, *69*, 645-656, doi:https://dx.doi.org/10.1007/s00011-020-01350-0.
31. Ma, P.; Zhang, C.; Huo, P.; Li, Y.; Yang, H. A novel role of the miR-152-3p/ERRFI1/STAT3 pathway modulates the apoptosis and inflammatory response after acute kidney injury. *Journal of Biochemical and Molecular Toxicology* **2020**, *34*, doi:10.1002/jbt.22540.
32. Ma, W.; Miao, X.; Xia, F.; Ruan, C.; Tao, D.; Li, B. The Potential of miR-370-3p and miR-495-3p Serving as Biomarkers for Sepsis-Associated Acute Kidney Injury. *Comput. math. methods med.* **2022**, *2022*, 2439509, doi:https://dx.doi.org/10.1155/2022/2439509.
33. Mao, H.; Huang, Q.; Liu, Y. MEG3 aggravates hypoxia/reoxygenation induced apoptosis of renal tubular epithelial cells via the miR-129-5p/HMGB1 axis. *Journal of biochemical and molecular toxicology* **2021**, *35*, e22649, doi:https://dx.doi.org/10.1002/jbt.22649.
34. Miller, D.; Eagle-Hemming, B.; Sheikh, S.; Joel-David, L.; Adebayo, A.; Lai, F.Y.; Roman, M.; Kumar, T.; Aujla, H.; Murphy, G.J.; Woźniak, M.J. Urinary extracellular vesicles and micro-RNA as markers of acute kidney injury after cardiac surgery. *Scientific reports* **2022**, *12*, 10402, doi:10.1038/s41598-022-13849-z.
35. Newbury, L.J.; Simpson, K.; Khalid, U.; John, I.; de Rivera, L.B.; Lu, Y.-A.; Lopez-Anton, M.; Watkins, W.J.; Jenkins, R.H.; Fraser, D.J.; Bowen, T. miR-141 mediates recovery from acute kidney injury. *Scientific reports* **2021**, *11*, 16499, doi:https://dx.doi.org/10.1038/s41598-021-94984-x.
36. Pavkovic, M.; Robinson-Cohen, C.; Chua, A.S.; Nicoara, O.; Cardenas-Gonzalez, M.; Bijol, V.; Ramachandran, K.; Hampson, L.; Pirmohamed, M.; Antoine, D.J.; et al. Detection of Drug-Induced Acute Kidney Injury in Humans Using Urinary KIM-1, miR-21, -200c, and -423. *Toxicol Sci* **2016**, *152*, 205-213, doi:https://dx.doi.org/10.1093/toxsci/kfw077.
37. Ramachandran, K.; Saikumar, J.; Bijol, V.; Koyner, J.L.; Qian, J.; Betensky, R.A.; Waikar, S.S.; Vaidya, V.S. Human miRNome Profiling Identifies MicroRNAs Differentially Present in the Urine after Kidney Injury. *Clinical Chemistry* **2013**, *59*, 1742-1752, doi:10.1373/clinchem.2013.210245.

38. Saikumar, J.; Hoffmann, D.; Kim, T.-M.; Gonzalez, V.R.; Zhang, Q.; Goering, P.L.; Brown, R.P.; Bijol, V.; Park, P.J.; Waikar, S.S.; Vaidya, V.S. Expression, circulation, and excretion profile of microRNA-21, -155, and -18a following acute kidney injury. *Toxicol Sci* **2012**, *129*, 256-267, doi:https://dx.doi.org/10.1093/toxsci/kfs210.
39. Shi, L.; Zhang, Y.; Xia, Y.; Li, C.; Song, Z.; Zhu, J. MiR-150-5p protects against septic acute kidney injury via repressing the MEKK3/JNK pathway. *Cell Signal* **2021**, *86*, 110101, doi:https://dx.doi.org/10.1016/j.cellsig.2021.110101.
40. Shihana, F.; Mohamed, F.; Joglekar, M.V.; Hardikar, A.A.; Seth, D.; Buckley, N.A. Urinary versus serum microRNAs in human oxalic acid poisoning: Contrasting signals and performance. *Toxicol Lett* **2020**, *334*, 21-26, doi:https://dx.doi.org/10.1016/j.toxlet.2020.09.003.
41. Sullo, N.; Mariani, S.; JnTala, M.; Kumar, T.; Woźniak, M.J.; Smallwood, D.; Pais, P.; Westrope, C.; Lotto, A.; Murphy, G.J. An Observational Cohort Feasibility Study to Identify Microvesicle and Micro-RNA Biomarkers of Acute Kidney Injury Following Pediatric Cardiac Surgery. *Pediatr Crit Care Med* **2018**, *19*, 816-830, doi:10.1097/PCC.0000000000001604.
42. Sun, J.; Cai, X.; Shen, J.; Jin, G.; Xie, Q. Correlation Between Single Nucleotide Polymorphisms at the 3'-UTR of the NFKB1 Gene and Acute Kidney Injury in Sepsis. *Genet Test Mol Biomarkers* **2020**, *24*, 274-284, doi:https://dx.doi.org/10.1089/gtmb.2019.0222.
43. Sun, S.q.; Zhang, T.; Ding, D.; Zhang, W.f.; Wang, X.l.; Sun, Z.; Hu, L.h.; Qin, S.y.; Shen, L.h.; He, B. Circulating Micro RNA-188,-30a, and-30e as Early Biomarkers for Contrast-Induced Acute Kidney Injury. *Journal of the American Heart Association* **2016**, *5*, e004138.
44. Tan, M.; Bei, R. Circ_0091702 serves as a sponge of miR-545-3p to attenuate sepsis-related acute kidney injury by upregulating THBS2. *J Mol Histol* **2021**, *52*, 717-728, doi:https://dx.doi.org/10.1007/s10735-021-09991-z.
45. Tang, B.; Li, W.; Ji, T.; Li, X.; Qu, X.; Feng, L.; Zhu, Y.; Qi, Y.; Zhu, C.; Bai, S. Downregulation of XIST ameliorates acute kidney injury by sponging miR-142-5p and targeting PDCD4. *J Cell Physiol* **2020**, *235*, 8852-8863, doi:https://dx.doi.org/10.1002/jcp.29729.
46. Wang, F.; Zhang, F.; Tian, Q.; Sheng, K. CircVMA21 ameliorates lipopolysaccharide (LPS)-induced HK-2 cell injury depending on the regulation of miR-7-5p/PPARA. *Autoimmunity* **2022**, *55*, 136-146, doi:https://dx.doi.org/10.1080/08916934.2021.2012764.
47. Wang, J.; Li, G.; Lin, M.; Lin, S.; Wu, L. microRNA-338-3p suppresses lipopolysaccharide-induced inflammatory response in HK-2 cells. *BMC Mol Cell Biol* **2022**, *23*, 60, doi:https://dx.doi.org/10.1186/s12860-022-00455-0.
48. Wang, M.; Wei, J.; Shang, F.; Zang, K.; Zhang, P. Down-regulation of lncRNA SNHG5 relieves sepsis-induced acute kidney injury by regulating the miR-374a-3p/TLR4/NF-κB pathway. *Journal of Biochemistry* **2021**, *169*, 575-583, doi:10.1093/jb/mvab008.
49. Wang, Q.-Y.; Zhang, R.-R.; Cui, L.; Sun, Y.-P. Silencing circ_0074371 inhibits the progression of sepsis-induced acute kidney injury by regulating miR-330-5p/ELK1 axis. *Mamm Genome* **2022**, *33*, 642-653, doi:https://dx.doi.org/10.1007/s00335-022-09961-0.
50. Wang, S.; Zhang, Z.; Wang, J.; Miao, H. MiR-107 induces TNF-alpha secretion in endothelial cells causing tubular cell injury in patients with septic acute kidney injury. *Biochem Biophys Res Commun* **2017**, *483*, 45-51, doi:https://dx.doi.org/10.1016/j.bbrc.2017.01.013.
51. Wei, Q.; Sun, H.; Song, S.; Liu, Y.; Liu, P.; Livingston, M.J.; Wang, J.; Liang, M.; Mi, Q.-S.; Huo, Y.; et al. MicroRNA-668 represses MTP18 to preserve mitochondrial dynamics in ischemic acute kidney injury. *J Clin Invest* **2018**, *128*, 5448-5464, doi:https://dx.doi.org/10.1172/JCI121859.
52. Xu, G.; Mo, L.; Wu, C.; Shen, X.; Dong, H.; Yu, L.; Pan, P.; Pan, K. The miR-15a-5p-XIST-CUL3 regulatory axis is important for sepsis-induced acute kidney injury. *Renal failure* **2019**, *41*, 955-966, doi:https://dx.doi.org/10.1080/0886022X.2019.1669460.
53. Xu, L.; Cao, H.; Xu, P.; Nie, M.; Zhao, C. Circ_0114427 promotes LPS-induced septic acute kidney injury by modulating miR-495-3p/TRAF6 through the NF-κB pathway. *Autoimmunity* **2022**, *55*, 52-64, doi:10.1080/08916934.2021.1995861.
54. Xu, Y.; Jiang, W.; Zhong, L.; Li, H.; Bai, L.; Chen, X.; Lin, Y.; Zheng, D. miR-195-5p alleviates acute kidney injury through repression of inflammation and oxidative stress by targeting vascular endothelial growth factor A. *Aging (Albany NY)* **2020**, *12*, 10235-10245, doi:https://dx.doi.org/10.18632/aging.103160.
55. Xun, L.; Li, Z.; Wang, H.; Zhang, P. THE VALUE OF COMBINING MIR-10A-5P LEVELS AND PLR TO EVALUATE THE PROGNOSIS OF SEPSIS PATIENTS WITH ACUTE KIDNEY INJURY. *Acta Medica Mediterranea* **2022**, *38*, 3303-3307, doi:10.19193/0393-6384_2022_5_488.

56. Yang, N.; Wang, H.; Zhang, L.; Lv, J.; Niu, Z.; Liu, J.; Zhang, Z. Long non-coding RNA SNHG14 aggravates LPS-induced acute kidney injury through regulating miR-495-3p/HIPK1. *Acta Biochim Biophys Sin (Shanghai)* **2021**, *53*, 719-728, doi:https://dx.doi.org/10.1093/abbs/gmab034.
57. Ye, J.; Feng, H.; Peng, Z. miR-23a-3p inhibits sepsis-induced kidney epithelial cell injury by suppressing Wnt/beta-catenin signaling by targeting wnt5a. *Braz J Med Biol Res* **2022**, *55*, e11571, doi:https://dx.doi.org/10.1590/1414-431X2021e11571.
58. You, T.; Kuang, F. CIRC_0008882 STIMULATES PDE7A TO SUPPRESS SEPTIC ACUTE KIDNEY INJURY PROGRESSION BY SPONGING MIR-155-5P. *Shock* **2023**, *59*, 657-665, doi:10.1097/SHK.0000000000002093.
59. Yuan, W.; Xiong, X.; Du, J.; Fan, Q.; Wang, R.; Zhang, X. LncRNA PVT1 accelerates LPS-induced septic acute kidney injury through targeting miR-17-5p and regulating NF-kappaB pathway. *Int Urol Nephrol* **2021**, *53*, 2409-2419, doi:https://dx.doi.org/10.1007/s11255-021-02905-8.
60. Zhang, B.; You, T.; Liu, Y.; Li, P. CIRC_0114428 INFLUENCES THE PROGRESSION OF SEPTIC ACUTE KIDNEY INJURY VIA REGULATING MIR-370-3P/TIMP2 AXIS. *Shock* **2023**, *59*, 505-513, doi:https://dx.doi.org/10.1097/SHK.0000000000002077.
61. Zhang, F.; Luo, X.; Wang, Y.; Ma, L.; Sun, D. LncRNA PMS2L2 Is Downregulated in Sepsis-Induced Acute Kidney Injury and Inhibits LPS-Induced Apoptosis of Podocytes. *Kidney Blood Press Res* **2023**, *48*, 515-521, doi:https://dx.doi.org/10.1159/000528053.
62. Zhang, H.; Che, L.; Wang, Y.; Zhou, H.; Gong, H.; Man, X.; Zhao, Q. Deregulated microRNA-22-3p in patients with sepsis-induced acute kidney injury serves as a new biomarker to predict disease occurrence and 28-day survival outcomes. *Int Urol Nephrol* **2021**, *53*, 2107-2116, doi:https://dx.doi.org/10.1007/s11255-021-02784-z.
63. Zhang, J.; Wang, C.J.; Tang, X.M.; Wei, Y.K. Urinary miR-26b as a potential biomarker for patients with sepsis-associated acute kidney injury: a Chinese population-based study. *Eur Rev Med Pharmacol Sci* **2018**, *22*, 4604-4610, doi:https://dx.doi.org/10.26355/eurrev_201807_15518.
64. Zhang, L.; Xu, Y.; Xue, S.; Wang, X.; Dai, H.; Qian, J.; Ni, Z.; Yan, Y. Implications of dynamic changes in miR-192 expression in ischemic acute kidney injury. *Int Urol Nephrol* **2017**, *49*, 541-550, doi:https://dx.doi.org/10.1007/s11255-016-1485-7.
65. Zhang, P.; Yin, J.; Xun, L.; Ding, T.; Du, S. CIRC_0002131 CONTRIBUTES TO LPS-INDUCED APOPTOSIS, INFLAMMATION, AND OXIDATIVE INJURY IN HK-2 CELLS VIA INHIBITING THE BINDING BETWEEN MIR-942-5P AND OXSR1. *Shock* **2023**, *60*, 517-524, doi:https://dx.doi.org/10.1097/SHK.0000000000002197.
66. Zhang, X.; Huang, Z.; Wang, Y.; Wang, T.; Li, J.; Xi, P. Long Non-Coding RNA RMRP Contributes to Sepsis-Induced Acute Kidney Injury. *Yonsei Med J* **2021**, *62*, 262-273, doi:https://dx.doi.org/10.3349/ymj.2021.62.3.262.
67. Zhao, H.; Chen, B.; Li, Z.; Wang, B.; Li, L. Long Noncoding RNA DANCR Suppressed Lipopolysaccharide-Induced Septic Acute Kidney Injury by Regulating miR-214 in HK-2 Cells. *Med Sci Monit* **2020**, *26*, e921822, doi:https://dx.doi.org/10.12659/MSM.921822.
68. Zheng, C.; Wu, D.; Shi, S.; Wang, L. miR-34b-5p promotes renal cell inflammation and apoptosis by inhibiting aquaporin-2 in sepsis-induced acute kidney injury. *Renal failure* **2021**, *43*, 291-301, doi:https://dx.doi.org/10.1080/0886022X.2021.1871922.
69. Zhou, F.; Liu, D.; Ye, J.; Li, B. Circ_0006944 aggravates LPS-induced HK2 cell injury via modulating miR-205-5p/UBL4A pathway. *Autoimmunity* **2023**, *56*, 2276066, doi:https://dx.doi.org/10.1080/08916934.2023.2276066.
70. Zhou, Y.; Qing, M.; Xu, M. Circ-BNIP3L knockdown alleviates LPS-induced renal tubular epithelial cell injury during sepsis-associated acute kidney injury by miR-370-3p/MYD88 axis. *J Bioenerg Biomembr* **2021**, *53*, 665-677, doi:https://dx.doi.org/10.1007/s10863-021-09925-0.
71. Zou, Y.-F.; Wen, D.; Zhao, Q.; Shen, P.-Y.; Shi, H.; Zhao, Q.; Chen, Y.-X.; Zhang, W. Urinary MicroRNA-30c-5p and MicroRNA-192-5p as potential biomarkers of ischemia-reperfusion-induced kidney injury. *Exp Biol Med (Maywood)* **2017**, *242*, 657-667, doi:https://dx.doi.org/10.1177/1535370216685005.
